# Health and economic impact of caregiving on informal caregivers of people with chronic diseases in sub-Saharan Africa: A systematic review

**DOI:** 10.1371/journal.pgph.0004061

**Published:** 2024-12-31

**Authors:** Ephraim Kisangala, Etheldreda Leinyuy Mbivnjo, Edward J. D. Webb, Barbara Barrett, Godfrey Zari Rukundo, Eve Namisango, Margaret Heslin

**Affiliations:** 1 Department of Health Service & Population Research, King’s College London, London, United Kingdom; 2 Department of Infectious Disease Epidemiology, London School of Hygiene and Tropical Medicine, London, United Kingdom; 3 Leeds Institute of Health Sciences, University of Leeds, Leeds, United Kingdom; 4 Department of Psychiatry, Mbarara University of Science and Technology, Mbarara, Uganda; 5 African Palliative Care Association, Kampala, Uganda; University College London, UNITED KINGDOM OF GREAT BRITAIN AND NORTHERN IRELAND

## Abstract

With a disproportionate burden of chronic diseases and severe shortage of health workers in sub-Saharan Africa, the region implicitly relies on informal caregivers (ICGs) to support the patients both within and outside the health facilities. The aim of this review is to systematically summarise evidence on the health and economic impact of caregiving on informal caregivers of patients with chronic diseases in sub-Saharan Africa. Medline (Ovid), CINAHL (EBSCOhost), PsycINFO (Ovid), Embase (Ovid), Global Health, and Web of Science databases were systematically searched to identify original articles that considered the economic and/or health impacts of caregiving in sub-Saharan Africa. The results from the included studies were synthesised narratively. After screening 4,951 records, 47 studies were included for synthesis. The articles were from all sub-regions of sub-Saharan Africa with more than half (25/47) of the studies focussing on caregivers for patients with cancer. Although the primary motivation for becoming caregivers was love and responsibility, the caring responsibilities described in twenty studies, had profound effects on the caregiver’s lives. Healthwise, the informal caregivers experienced changes in their physical and mental health like developing musculoskeletal problems and depression. Economically, caregiving was expensive, and financially draining. The opportunity cost of caregiving included loss of jobs, loss of income, foregoing planned important activities and missed education opportunities. Informal caregivers reported a range of mainly negative health and economic effects of the work they do. Health care systems should consider how to better support caregivers in terms of their own physical and mental wellbeing. Also, governments should develop strategies to financially support informal caregivers.

## Introduction

Global increases in healthcare expenditure have not been proportional across the world [1]. In sub-Saharan Africa, government spending on health is very low whilst out-of-pocket expenditure is significantly high. These countries do not have well-developed health insurance systems and their out-of-pocket health expenditure is sometimes higher than 70% of the respective national health expenditures [2,3]. Universal health insurance is still in its infancy in sub-Saharan Africa, with only four countries having at least 20% of their populations covered by the government-funded health insurance [3,4]. Therefore, the family (informal caregiver(s)) of a person with a chronic disease in this region is often faced with a double burden of the long-term nature of the disease and the limited availability of diagnostic and disease management services [5]. Chronic diseases which are projected to be the leading cause of morbidity and mortality in Africa by 2030 are slow-progressing diseases that last long in the body and cannot be transmitted from one person to another [6,7].

The term informal caregiver is used to refer to any layperson who provides regular emotional, physical, financial, and medical support to the sick or to people with disabilities, often without expectation of an immediate reward [8,9]. Throughout this review, ‘informal caregiver’ and ‘caregiver’ are used interchangeably to mean the same thing unless otherwise specified. Many times, informal caregivers have a familial relationship with the care recipient, often assuming the caregiving role as parents, spouses, children, siblings, or other relatives. These caregivers are predominantly female and frequently take on the caregiving role with minimal or no preparation [10]. It is understood that the caregivers in sub-Saharan Africa perform numerous tasks, some of which are typically responsibilities of healthcare professionals in other parts of the world [10,11]. They perform these activities whenever required, whether day or night, at home or in the hospital and under any situation. For example, caregivers sometimes sleep on hospital floors during the night to provide companionship and maintain close proximity to the care recipient in order to address any immediate concerns required by either the patient or the medical personnel at the hospital [12]. Accordingly, they are faced with unique challenges, such as overwhelming responsibilities, high out-of-pocket health expenditure, stigma, and limited access to healthcare services [10,11].

Over the years, reviews have been conducted to assess the impact of caregiving in sub-Saharan Africa. The focus of these reviews has been on specific conditions such as HIV [13], cancers [10], old age, end-of-life care [14], mental health disorders [15] and stroke. Other researchers limited their reviews to studies within specific countries [16] or specific settings like hospitals [17].

In this review, we aimed to systematically summarise evidence on the health and economic impact of caregiving on caregivers of patients with chronic diseases in the whole of sub-Saharan Africa. This was achieved through the following three objectives: 1) To identify the activities caregivers of people with chronic diseases in sub-Saharan Africa perform 2) To synthesize the reasons why caregivers of people with chronic diseases in sub-Saharan Africa took on the caregiving roles 3) To summarise evidence on the health and economic impact of caregiving on caregivers of people with chronic diseases in sub-Saharan Africa. From our understanding, this is the first review to explore how caregiving affects the health and economic aspects of caregivers of people with major chronic diseases in the whole of sub-Saharan Africa.

## Methods

This systematic review was conducted, adhering to the guidelines laid out in the Preferred Reporting Items for Systematic Reviews and Meta-Analyses (PRISMA) statement [18]. The protocol for this review was registered with PROSPERO under the registration number of CRD42022358531. The databases were searched between September and October 2022, and updated in May 2024.

### Eligibility criteria

The criteria for selecting studies were as follows:

#### Study design

All original studies, irrespective of the design were included in this review. These included randomised controlled trials, case-control, cohort, cross-sectional and qualitative studies. However, commentaries and reviews were excluded.

#### Population

Studies with informal caregivers providing care to people with chronic diseases were included. We included the major chronic diseases which are known to cause significant morbidity and over 80% of premature deaths ‐ these were cardiovascular diseases, chronic obstructive pulmonary diseases, cancers and Type 2 diabetes [19]. We excluded mental health conditions because there is already a systematic review on the impact of providing informal care to people with mental health conditions in sub-Saharan Africa [15]. We also excluded informal caregivers of people with a co-morbidity that is not a major chronic disease. We did not include studies that had less than half (50%) of informal caregivers in active caregiving roles. Caregivers may not actively provide care when the care recipient has significantly improved, died and when another person has taken over the caring role. We also excluded studies where results for the population of interest were pooled with others (such as professional caregivers, paid caregivers, and volunteers) and if the proportion of the population of interest was under 50%. Such studies would dilute the relevance of the findings and make them less applicable to informal caregivers, who are this review’s population of interest. Where results were presented separately for the population of interest, we included the studies regardless of what proportion of the total sample they made up.

#### Outcomes

The studies that were selected for inclusion in this review had findings with information about how caregiving affected the health or economic aspects of the informal caregiver’s life. Such information had to be provided in the findings.

#### Setting

Studies were selected if they were conducted within sub-Saharan Africa as defined by World Bank (https://openknowledge.worldbank.org/pages/focus-sub-saharan-africa). The search was not restricted to any language since articles published in languages other than English were translated.

### Information sources

EK searched for relevant original articles from the following databases between September and October 2022, and updated in May 2024: Medline (Ovid), CINAHL (EBSCOhost), PsycINFO (Ovid), Embase (Ovid), Global Health and Web of Science. The searches were conducted from the inception of each database up to the date of each search. Full details of the searches including the databases, search strategy and the search outcomes can be found in **S1 File.**

In addition, the reference lists of selected articles and relevant systematic review reports were searched. Lastly, primary authors of editorial letters, abstracts from conference proceedings, abstracts without full-text articles and commentaries identified through the searches were contacted by email for full-text original articles.

### Search strategy

The search strategy development process involved identifying candidate search terms by reading the titles, abstracts, keywords, and search strategies in the three known related articles [17,20,21]. A draft search string was formed by appropriately combining the identified search terms and MeSH terms using Boolean operators. The search terms: Informal caregiver, sub-Saharan Africa, and Chronic Diseases, were combined using the Boolean operator “AND,” while the thesauruses and MeSH words for each search term were combined using “OR.” This was improved after a review and discussion with two senior researchers (BB and MH) and a librarian from King’s College London, all of whom have experience in systematic reviews. This was done to enhance suitability of search terms and to ensure the search strategy would find all the relevant literature. The final search string as used in each database can be found in **S1 File.**

### Selection process

The articles that were retrieved from all the searched databases were exported to Endnote (version 20) for removal of the duplicate articles. This was done by the first reviewer (EK). The remaining articles were then transferred to Rayyan software (https://www.rayyan.ai/) where title and abstract screening was done. The articles that did not meet the agreed criteria were excluded while those that potentially met the eligibility criteria were included for full-text screening. Full-text screening was carried out for the articles whose full-text versions were retrieved. The articles whose full-texts were unavailable even after contacting the primary authors were excluded. At each stage, the screening was independently conducted by two reviewers. While EK screened all the articles in both stages, the second reviewer (ELM) screened a random 20% of the articles at the title and abstract screening stage, and a random 25% during the full-text screening. There was 96% agreement between the two reviewers and all disagreements were resolved by a discussion with input from other authors. In this review, all non-English papers were translated into English using Google Translate, a choice made due to its known reliability and accuracy in English with 91% agreement between native language reviewers and reviews who use google translate (range, 85% to 97%) [22]. EK confirmed that the selected articles were eligible for inclusion in this study.

### Quality of the included studies

The quality of the articles was independently assessed by two reviewers (EK and ELM) using the Mixed Methods Appraisal Tool, a critical appraisal tool that has been tested and found to be reliable and efficient [23,24]. It is used to assess the quality of studies of different designs including qualitative and quantitative studies. For each article (depending on the study design), there are five (5) questions that require a response of Yes, No or Can’t Tell. In this review, the Yes was given a score of 1 and No or Can’t Tell was given a score of 0. Any disagreement was resolved by consensus.

### Data extraction and analysis

Relevant data was extracted from all articles by EK and entered into a data extraction form, developed with the study objectives in mind. The data that was extracted into the form included; the name of the first author of the study, the year of publication, the country where the study was conducted, study design, sample size, participants’ age and sex, their marital and employment status, the relationship of informal caregiver to the patient, caregiving activities, setting (home or hospital setting), type of chronic disease, scale used to measure impact, the health and economic impact of caregiving on informal caregivers and their motivation to provide care. Thereafter, data were synthesized narratively.

## Results

### Study selection

**Fig 1:**
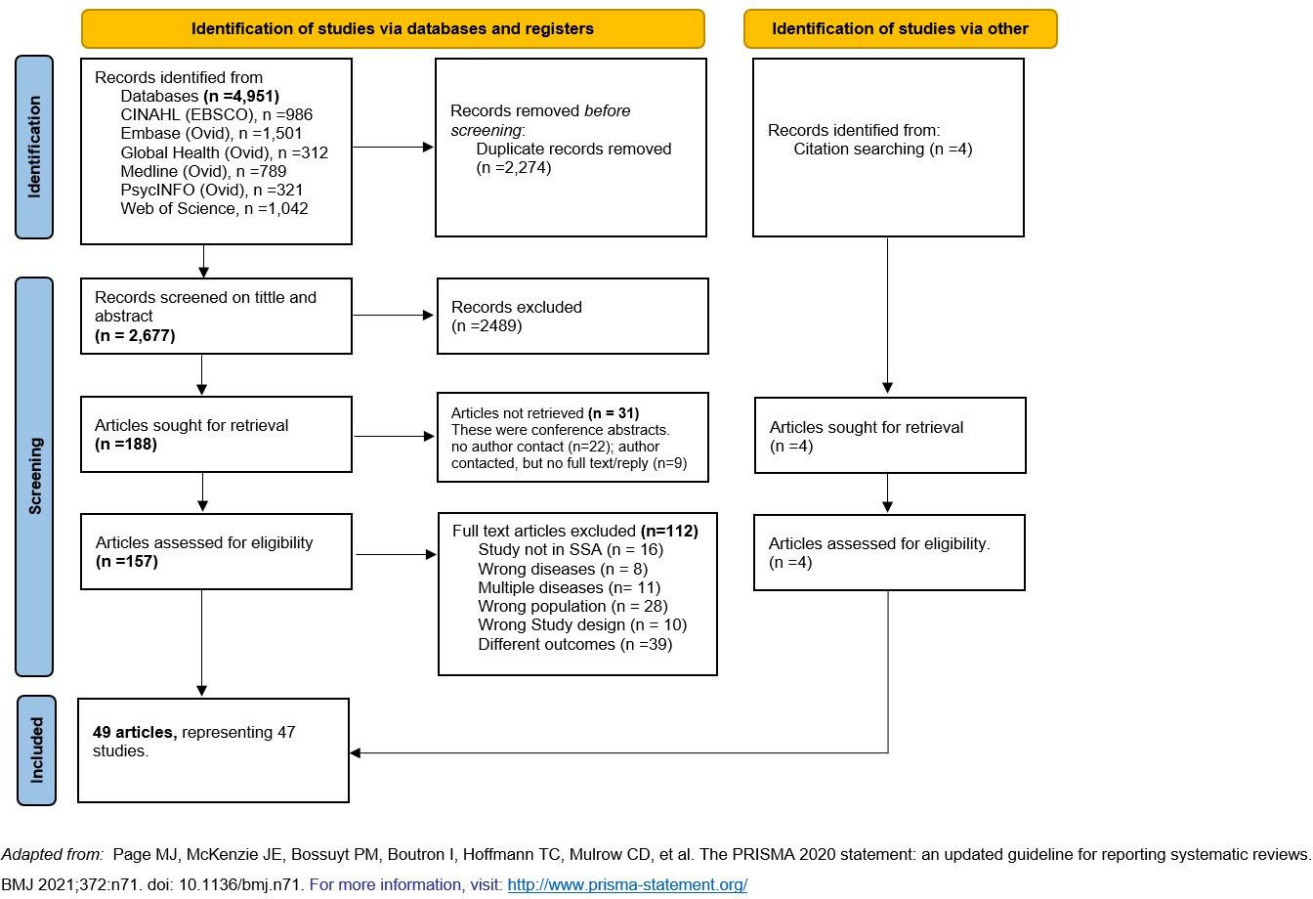
PRISMA flow diagram of the screening and selection process in this review.

As shown in Fig 1 (PRISMA diagram), 4,951 records were retrieved through a systematic search of seven databases and a targeted search of the reference list of the already identified relevant articles (4). After removing 2,274 duplicate articles, 2,489 articles were excluded during the title and abstract screening and another 31 articles were also excluded because they were conference abstracts whose full-text articles could not be retrieved. During the full-text screening, 112 articles were excluded for various reasons including being conducted outside the sub-Saharan African region (n = 16), focusing on conditions other than chronic diseases (n = 8), having groups of participants with other diseases (n = 11), having participants that were paid or professional caregivers (n = 28), writing the article as a review or commentary (n = 10) and having outcomes that are not related to health or economic impact of caregiving (n = 39). The remaining 49 articles representing 47 studies were included in the review. This is because four articles, two from South Africa [25,26] and two from Ghana [27,28], were from one study in each of these countries.

### Study characteristics

All studies were written in English except one that was in French [29]. The authors used qualitative methods in 22 of 47 studies. Twenty-four studies used quantitative methods while mixed-methods approach was used in one study. Of the quantitative studies, three [30–32] were longitudinal studies and the rest had a cross-sectional study design. The studies were from fourteen countries representing all the sub-regions of sub-Saharan Africa. Nigeria and South Africa had the highest number of studies in this review, with eleven [33–43] and ten [11,25,26,30,31,44–49] studies respectively, while each of the following countries had just one (1/47) study; Togo [50], Zimbabwe [51], Kenya [52], Sudan [53], Democratic Republic of Congo [54] and Cameroon [29]. The participants in the selected studies were drawn from various settings, including outpatient facilities [29,31,33,34,36–40,42,44,49,51,52,54–56], (n = 17) inpatient facilities [12,21,30,57–59] (n = 6) both outpatient and inpatient facilities [25,26,60–62] (n = 4), homes [32,46] (n = 2) and hospice centres [47] (n = 1). The setting was not specified in seventeen studies [9,11,27,28,35,41,43,45,48,50,53,63–69]. See Table 1.

**Table 1:** Summary of the characteristics of the reviewed studies.

Author, year, Country	Study design (Setting)	Participants	Sample size	Disease	Relationship to Patient	Duration of caring	Time spent in care	Instrument used to assess impact	Heath and economic impact of caregiving
Yusuf (2011) [33] Nigeria	Quantitative (Health facility outpatients)	Family caregivers Mean age: 57 years Age range: Not reported Sex: Female, 43 (42%) **Employment:** Unemployed 25 (24.3%) Employed 78 (75.7%)	103	Cancers (Not specified)	Spouse: 3 (2.9%) Son/daughter: 74 (71.9%) Sibling: 26 (25.2%)	Not reported	Not reported	General Health Questionnaire (GHQ-30), Zarit Burden Interview (ZBI)	• Mean ZBI score was 29.16; mean GHQ score was 3.67 • Psychological morbidity in 48 (46.6%) caregivers
Wassie (2022) [57] Ethiopia	Quantitative (Health facility inpatients)	ICGs of adults Mean age: 35 years Age range: Not reported Sex: Female, 176 (42.72%) **Employment:** Employed 282 (68.44%)/Unemployed 130 (31.56%)	412	Breast cancer 91 (22.09%); Cervical cancer 54(13.11%); Gastric cancer 30(7.28%); Others 237 (57.52%)	Spouse 140 (33.98%); Child 150 (36.41%); Siblings 85 (20.63%); Parents 21 (5.1%); Other relatives 16 (3.88%);	more than 2 weeks	Not reported	Patient Health Questionnaire-9 (PHQ-9) for Depression	Prevalence of depression was 45.15% (95% CI: 40.38–50.001)
Walubita (2018) [12] Zambia	Qualitative (Health facility inpatients)	**ICGs of children with cancers** Mean age: Not reported Age range: 20 to 58 years Sex: Female, 14 (93.3%) **Employment:** Not reported	15	Cancer of the kidney (4), Leukaemia (3), Retinoblastoma (3), Lymphoma (2), Cancer of the liver (1), Cancer of the lungs (1)	Parent 12; Grandparent 2; Great grandparent 1;	Not reported	Not reported	None	• Loss of jobs/source of income. • Reduced economic productivity
Vincent-Onabajo (2018) [34] Nigeria	Quantitative (Health facility outpatients)	ICGs of people with stroke Mean age: 33.2 years Age range: 19 to 60 years Sex: Female, 55 (61.1%) **Employment:** Unemployed 60 (66.7%)/Employed 30 (33.3%)	90	Stroke	Offspring 53 (58.9%); Spouse 22 (24.4%); Sibling 15 (16.7%).	Not reported	Not reported	Nordic Musculoskeletal Questionnaire (NMQ)	• 74 (82.2%) caregivers experienced musculoskeletal symptoms (mms) in last 7 days. These symptoms were distributed as follows, 72% had Lower back symptoms, 40% upper back, 24.4% shoulder, 3.3% wrist. • Other mms symptoms reported were on the neck, hips, buttocks, thighs, knees, ankle and feet and elbow joint.
Thomas (2008) [44] South Africa	Qualitative (Health facility outpatients)	Primary ICGs Mean age: 47.2 years Age range:16 to 67 years Sex: Female 4 (66.7) Employment: Not reported	6	Stroke	Husbands 2 (33.3); Wife 2 (33.3%); Daughters 2 (33.3%)	Not reported	Always with the care recipient	None	• Resigned from work • Financial strain • Missed education (attending school) • Frequent infection due to low immunity coming from stressDepression
Tchokote (2020) [29] Cameroon	Qualitative (Health facility outpatients)	Primary or secondary ICGs Mean age: 32.75 years Age range: 20 to 50 years Sex: Female 2 (50%) Employment: Employed 1 (25%); Unemployed 3 (75%)	4	Stroke	Grandchildren 2 (50%); Wife 1 (25%); Daughter 1 (25%)	Not reported	Not reported	None	• Support with transport costs • Emotional reaction to diagnosis • Psychological exhaustion from heavy work • Depression • Less sleep • Less time for work • Less time for school
Serfontein (2019) [30] South Africa	Quantitative (Rehabilitation centre inpatients)	Primary ICGs Mean age: Not reported Age range: 23 to 76 years Sex: Female 50 (79.4%) Employment: Employed 30 (47.6%); Unemployed 33 (52.4%)	63	Cerebrovascular accident (CVA)	Spouses 32 (50.8%); Children 18 (28.6%); Other relatives (13 20.6%)	≥ 2 months	13.2% (5) spend ≥4 hours/day 55.6% (35) spend 2–4 hours/day & 23.8% (15) spent 5–6 hours/day	Modified Caregiver Strain Index (MCSI), & Interpretation guide of the CSI	• ICGs (28.6%) experienced work-related adjustments including changing to flexible working hours, taking night shifts and working from home. • ICGs were financially strain either regularly (n = 23; 36.5%) or at times (n = 17; 27%).
Scheffler (2019) [31] South Africa	Quantitative (Health facility outpatients)	**ICGs [*73/80 (91*.*2%)-ICGs; & 7/80 (8*.*8%) paid carers]*** Mean age: 47.4 years, Age range: 12 to 79 years Sex: Female 73 (82.0%) Employment: Not reported	89	Stroke	Spouse/partner 25 (28.4%) Son/daughter 33 (37.5%) Other family 19 (21.6%) Friend 6 (6.8%) Other 6 (5.7%)	Not reported	Not reported	Caregiver Strain Index	Financial strain at: • Baseline 30/84 (35.7%), & • Final assessment 25/66 (37.9%)
Owoo (2022) [28] Ghana	Qualitative (Not specified)	Family caregivers Mean age: 48.67 years Age range: 27 to 67 years Sex: Female 11 (91.67%) Employment: Employed: 9 (75%); Unemployed: 3 (25%)	12	Prostate cancer	Father 6 (50%) Husband 6 (50%)	1 year 8 (66.67%) 2 years 3 (25%) 6 months 1 (8.33%)	Not reported	None	• Lack of adequate sleep and lack of sleep • Worsening of pre-existing conditions • Pain • Altered eating patterns and habits causing weight loss
Owoo (2022) [27] Ghana	Qualitative (Not specified)	Family caregivers Mean age: 48.67 years Age range: 27 to 67 years Sex: Female 11 (91.67%) Employment: Employed: 9 (75%); Unemployed: 3 (25%)	12	Prostate cancer	Father 6 (50%) Husband 6 (50%)	1 year 8 (66.67%) 2 years 3 (25%) 6 months 1 (8.33%)	Not reported	None	Paid medical bills despite financial constraints
Onyeneho (2021) [35] Nigeria	Quantitative (Not specified)	Primary caregiver Mean age: 37.68 YEARS Age range: 10 to >51 years Sex: Female 11 (91.67%) Employment: Employed: 9 (75%); Unemployed: 3 (25%)	182	Cancer (Not specified)	Brother 12 (6.6%) Sister 20 (11.0%) Parents 119 (65.4%) Husband 17 (9.3%) Wife 14 (7.7%)	<1 month 52 (28.6%) 1 month to 6 months 63 (34.6%) >6 months to 1 year 21 (11.5%) >1 year 46 (25.3%) Mean±SD 2.34±1.14	Not reported	Zarit Burden Interview (ZBI)	• The mean score of the perceived financial effects of caregiving experienced by caregivers is 2.14, (> threshold of 2.00) • Causes of financial strain: • Lack of money (mean = 2.59 > 2.14), • Jobs affected (mean = 2.86 > 2.14), • Loss of money (spent) (mean = 2.21 > 2.14).
Okeke (2020) [36] Nigeria	Quantitative (Health facility outpatients)	Informal caregivers Mean age: 41.1 years Age range: 18 to 75 years Sex: Female 179 (57.6%) Employment: Not reported	311	Stroke	Parents 100 (32.1%) Children 18 (5.8%) Spouse 106 (34.1%) Non relatives 18 (5.8%) Other relatives 69 (22.2%)	1 to 3 months 112 (36%) 4 to 6 months 26 (8.3%) 7 to 12 months 22 (7.1%) 13 to 24 months 32 (10.3%) More than 24 months 119 (38.3%)	Not reported	Zarit Burden Interview (ZBI); & Patient Health Questionnaire (PHQ-9)	• ICGs were depressed (mild depression 93 (29.9%), moderate depression 48 (15.4%), moderately severe depression 28 (9.0%) & severe depression 16 (5.1%). • Association between burden of care and depression (χ2 = 99.3, p < 0.001);, • There was a positive correlation between the ZBI scores for the burden of care and the PHQ9 scores for depression in ICGs (r2 = 0.751; p<0.001), • There was a positive correlation between severity of depression and burden of care (r2 = 0.68, p < 0.001).
Ohaeri (1999) [37] Nigeria	Quantitative (Health facility outpatients)	Informal caregivers Mean age: 35.6 Age range: 18 to 75 years Sex: Not reported Employment: Employed 52 (70.4%), Unemployed 21 (29.6%);	73	Cervix cancer Breast cancer	Husband 24 (32.9%) Child 36 (49.3%) Others: 13 (17.8%)	Not reported	Daily contact with patient 55 (75.3%)	Burden questionnaire	• Moderate/major loss of revenue by 31.6% ICGs • Expenditure on patient had major effect on 68.4% ICGs. • Major/moderate loans taken by 23.3% ICGs.
Ogunmodede (2019) [38] Nigeria	Quantitative (Health facility outpatients)	*Primary caregivers [1(1%) paid carers*, *99 (99%) ICGs]* Mean age: 46.5 years Age range: ≤20 to >60 years Sex: Female 56 (56.0%) Employment: Employed 66 (66.0%), Unemployed 34 (34.0%)	100	Type 2 Diabetes Mellitus	Parents 10 (10.0%) Child 40 (40.0%) Spouse 34 (34.0%) Siblings 11 (11.0%) Unrelated 1 (1.0%) Others 4 (4.0%)	Not reported	Daily contact 83 (83.0%) Weekly contact 10 (10.0%) 2–3 times a month 5 (5.0%) Several times a year 2 (2.0%) **Number of contact hours** </ = 5hours: 4 (4.0%) 6–10 hours: 18 (18.0%) 11–15 ours 22 (22.0%) >16 hours 31 (31.0%)	Zarit Burden Interview (ZBI) and General Health Questionnaire (GHQ-12)	35% of caregivers had GHQ ≥ 3 indicating psychological distress.
Muriuki (2023) [52], Kenya	Quantitative (Health facility outpatients)	Family caregivers Mean age: Not reported Age range: 18 to >61 years Sex: Female 147 (57.6%) Employment: Employed 109 (42.8%), Unemployed 146 (57.3%)	255	Cancer (not specified)	Not reported	at least 2 weeks	Not reported	Modified Caregiver Strain Index (MCSI) tool	Prevalence of ICG with: • Financial strain was100% • Disturbed sleep was 56.5%Work related adjustments was 72.2%
Muliira (2019) [60] Uganda	Quantitative (Outpatients and inpatients)	Family caregivers Mean age: 36 years Age range: 18 to >51 years Sex: Female 208 (73.2%) Employment: Employed 161 (56.7%), Unemployed 123 (43.3%)	284	Breast cancer (18.3%), cervix cancer (12.3%), and leukaemia (12.3%) and others (cancers of oesophagus, prostate, ovary, pancreas, stomach, Lymphomas, and multiple myeloma).	Spouse 57 (20.1%) Child 110 (38.7%) Others 117 (41.2%)	7–11 months 135 (47.5%) 12–24 months 91 (32.1%) ≥25 months 58 (20.4%)	≤48 hours/week 57 (20.1%) 49–120 hours/week 92 (32.4%) ≥121 hours/week 135 (47.5%)	Hospital Anxiety and Depression scale, Caregiver Reaction Assessment scale	• The ICGs experienced (prevalence): • Clinically significant symptoms of anxiety (35.2%) • Severe symptoms of anxiety (20%) • Clinically significant symptoms of depression (48.2%) • Severe symptoms of depression (27.5%)
Mthembu (2016) [45] South Africa	Qualitative (Not specified)	Informal caregivers Mean age: 58.5 years Age range: 24 to 79 years Sex: Female 5 (83.33%) Employment: Not reported	6	Lung Disease Hypertension Stroke Diabetes mellitus	Spouse 2 (33.33%) Children 3 (50%) Other relatives (Daughter in Law) 1 (16.67%)	Not reported	Not reported	None	• Work-related challenges eg loss of jobs, struggle to balance jobs, education and care’ • Struggled to raise money for medicationGained emotional strength from caring.
Mlaba (2021) [26] South Africa	Qualitative (Outpatients and inpatients)	Informal caregivers (*patients of 3 (15%) of IGCs had died*) Mean age: 55.05 years Age range: 21 to 84 years Sex: Female 14 (70.0%) Employment: Not reported	20	Various cancers (Not specified)	Spouse 7 (35.0%) Offspring/children 5 (25.0%) Parent 1 (5.0%) Other relatives 7 (35.0%)	Not reported	Not reported		• Psychological distress. • Disrupted sleep patterns
Mlaba (2020) [25] South Africa	Qualitative (Outpatients and inpatients)	Informal caregivers (*patients of 3 (15%) of IGCs had died*) Mean age: 55.05 years Age range: 21 to 84 years Sex: Female 14 (70.0%) Employment: Not reported	20	Various cancers (Not specified)	Spouse 7 (35.0%) Children 5 (25.0%) Parent 1 (5.0%) Other relatives 7 (35.0%)	Not reported	Not reported	None	• Jobs affected through less work time, losing jobs, changing jobs to one closer to patient. • High medical and food expenses. • Loss of income due to affected jobs and high medical expenses. • They became less financially stable than before.
Mensah (2021) [63] Ghana	Qualitative (Not specified)	Husbands of women with advanced breast cancer Mean age: Not reported Age range: 34 to 55 years Sex: Female 0 (0%) Male 15 (100%) Employment: Employed 15 (100%)	15	Breast cancer	Husband 15 (100%)	4 to 11 months	Not reported	None	• Employment challenges (failure to balance work and care, inability to meet job expectations, lateness to work, and absenteeism). They were questioned, warned or dismissed from work or their businesses failed. • Loss of income and social networks • Experienced debt and financial constraints • Sold off valuable family property to meet medical expenses/requirements • Developed psychological problems eg identity crisis
Mekonnen (2020) [64] Ethiopia	Mixed (Not specified)	Parents of children with cancer Quantitative Mean age: Not reported Age range: 18 to >49 years Sex: Female 145 (52.7%) Employment: Not reported Qualitative Mean age: Not reported Age range: 36–45 years Sex: Female 13 (65%) Employment: Not reported	295 (Quantitative = 275; Qualitative = 20)	Cancer (Not specified)	Quantitative Parents 275 (100%); and Qualitative Parents 20 (100%)	Qualitative (n = 275) 1–3 weeks 14 (5.1%) 4–6 weeks 148 (53.8%) 7–9 weeks 64 (23.3%) ≥10 weeks 49 (17.8%)	Not reported	Likert-type scale questionnaire adopted from Beck’s depression inventory scale	• Financial problems • They made less and spend more money • ICGs worried about the high treatment costs. • They’re psychologically traumatised.Prevalence of depression among the ICGs of children with cancer was 49.1% (mild mood disturbance), 7.3% (borderline clinical depression, 6.2% (moderate depression), 6.5% (severe depression) and 3.3% (extreme depression).
Masuku, (2018) [46] South Africa	Qualitative (At their home)	Primary caregivers Mean age: 38 years. Age range: 21 to 65 years Sex: Female 14 (100%) Employment: Employed 6 (42.9%), Unemployed 8 (57.1%)	14	stroke	Children 5 (35.7%) Other relatives 1 (7.1%) Wife 6 (42.9%) Sister 2 (14.3%)	RANGE (3 months to more than 3 years) </ = 6 months 6 (42.9%) 7 months to </ = 12 months 3 (21.4%) >12 months to </ = 24 years 3 (21.4%) 24 months to 48 months 2 (14.3%)	Not reported	None	ICGs experienced: • Lifestyle adjustments to accommodate the extra burden caused by care. • Missed treatment & reviews due to lack of money and poverty. • Emotional and psychological challenges • Poor health especially the older caregivers • Change of jobs to work near home.
Masika (2020) [58] Tanzania	Qualitative (Health facility inpatients)	Guardians of children Mean age: 38 years Age range: Not reported Sex: Female 15 (68.2%) Employment: Not reported	22	leukemia (5), eye cancer (5), cancer of the glands (5), kidney cancer (2), stomach cancer (1), thyroid cancer (1), and liver cancer (1)	Guardian 22 (100%)	Not reported	Not reported		• They were financial concerns due to prioritisation of care demands and costs over their economic activities, • Separation from their families during care. • Sold off family property to meet medical bills • Experienced emotional stress • Lack of support from family, health workers or community. • Expressed need to generate an income while in hospital
Marima (2019) [51] Zimbabwe	Quantitative (Health facility outpatients)	ICGs Mean age: 41.5 years Age range: Not reported Sex: Female 50 (70.4%) Employment: Not reported	71	Stroke	Not reported	Median (IQR) 4 (2–13) months; Most had provided care for at least 4 months	Not reported	Shona Symptoms Questionnaire (SSQ); Multidimensional Scale of Perceived Social Support (MSPSS)	45.1% of the caregivers showed excessive psychiatric morbidity. The median SSQ score was 7 (IQR: 3–9)
Maree (2018) [11] South Africa	Qualitative (Not specified)	Primary ICGs Mean age: 41.7 years Age range: 20 to 65 years Sex: Female 10 (50%) Employment: Not reported	20	various cancers (Not specified)	Children: 10 (50%) Spouse: 4 (20%) Friends: 3 (15%) Other relatives: 2 (10%) Sibling: 1 (5%)	Between 3 weeks and 10 years, with an average of 24.8 months (SD ± 26.9).	Not reported		ICGs work was affected through. • Loss of jobs • No time to search/apply for jobs • Absenteeism at work • Financial hardship worsened by the transport costs • Increased responsibilities.
Kitoko (2022) [54] Democratic Republic of Congo	Quantitative (Rehabilitation centre Outpatients)	ICGs Mean age: 42.3 years Age range: Not reported Sex: Female 58 (68.2%) Employment: Employed: 54 (63.5%); Unemployed: 31 (36.5%)	85	Stroke	Not reported	At least 3 months	Not reported	Zarit Burden Inventory (ZBI); The Hospital Anxiety and Depression Scale (HADS); The modified Rankin scale (mRS).	• Anxiety • Yes: (HADSA >7) 28 (32.9%) • No: (HADSA ≤ 7) 57 (67.1%) • Mean Score: 5.85 ± 3.86 • Depression • Yes: (HADS D>7) 30 (35.3%) • No: (HADS D≤ 7) 55 (64.7%) • Mean Score: 6.15 ± 4.39 • Mixed type (Anxiety and Depression) • Yes: (HADS >14 28 (32.9%) • No: (HADS ≤ 14) 57 (67.1%) • Mean Score: 11.88 ± 7.12 • Using ZBI scores: 84.7% of the caregivers had (perceived) a high burden of depression
Khondowe (2007) [55] Zambia	Qualitative (Health facility outpatients)	Caregivers of stroke outpatients	10	Stroke	Not reported	Not reported	Not reported		• Balancing work and care was difficult. • Money was inadequate for patient care and family. • Money was used to transport patients and getting extra support during care.
Ketlogetswe (2022) [47] South Africa	Qualitative (Hospice inpatients)	Primary ICGs Mean age: 52 years Age range: 23 to 84 years Sex: Female 19 (86.36%) Employment: Employed: 10 (45.45%); Unemployed: 12 (54.54%)	22	cancers of head, neck, prostate, thyroid, breast, lung, vulva, & blood	mostly mothers of patient, others included partners, sons, daughters and a neighbour (No statistic given)	13 months on average	Not reported		• ICGs were fearful, anxious and emotionally distressed etc about possible death of patient • Care was exhausting and demanding causing fatigue and sleep deprivation • They got health challenges, like pain, headaches, backache, fatigue and hypertension • Caregiving was financially draining eg buying prescribed medicines • Caregivers lost their jobs.
Katende (2017) [61] Uganda	Quantitative (Outpatients and inpatients)	Family caregivers Mean age: 33 years Age range: 18 to 60 years Sex: Female 80 (67.2%) Employment: Employed: 73 (61.3%) Unemployed: 46 (38.7%)	119	Cancer (Not specified)	Parent: 32 (26.9%) Other 1st degree relatives: 49 (41.2%) Extended family: 32 (26.9%) Other: 6 (5.0%)	Not reported	Not reported	Hospital Anxiety and Depression Scale (HADS)	• 45% participants had abnormal levels of anxiety (ALA). Male carers were most affected, and had higher levels of anxiety than their female carers (46.2% v.43.8%). • 26% caregivers had abnormal levels of depression (ALD). Males more affected than females and had higher levels of depression (30.8% v. 21.3%)
Jones (2012) [32] Tanzania	Quantitative (At their home)	Primary ICGs of physically dependant stroke survivor Mean age: 47 years Age range: 16 to 73 years Sex: Female 23 (85.19%) Employment: Employed: 17 (63.00%), Unemployed: 10 (37.00%)	27	Stroke	Children: 14 (51.85%) Spouse:11 (40.74%) Grand children: 2 (7.41%)	At least 6 months	Not reported	Hospital anxiety and depression scale (HAD)	• 22.2% of caregivers experienced anxiety (14.8% had mild, 3.7% had moderate and 3.7% had severe anxiety). • 40.7% caregivers had depression (29.6% had mild, 7.4% had moderate and 3.7% had severe depression)
Gertrude (2019) [21] Uganda	Qualitative (Health facility inpatients)	ICGs Mean age: Not reported Age range: 18 to ≥55 years Sex: Female 14 (56%) Employment: Employed: 21 (84%%), Unemployed: 4 (16%)	25	STROKE: Ischaemic stroke: 15 (60%) Haemorrhagic stroke: 8 (32%) Stroke (type Missing): 2 (8%)	Spouse: 9 (36%) Child/Child in law: 9 (36%) Other: 7 (28%)	4 months to 1 year: 10 (40%) > = 1 year: 15 (60%) (Median duration of care:16 months)	Not reported	None	• High financial expenditure during care. • Difficulty balancing job, family and caregiving responsibilities. • Lost jobs in order to provide care, leading to financial strain. • Physical health complaints (body pain, swollen feet, and fatigue) • Psychological distress and grief following loss of the patient.
Gbiri (2015) [39] Nigeria	Quantitative (Physiotherapy clinic outpatients)	ICGs assisting with ADL Mean age: 39.2 years Age range: 17 to 74 years Sex: Female 76 (48.4%) Employment: Employed: 105 (66.9%); Unemployed: 52 (33.1%)	157	Haemorrhagic stroke 61 (38.9%) Ischaemic stroke 96 (61.1%)	Children: 75 (47.8%) Spouse:45 (28.7%) Children: 21 (13.4%) Significant others: 16 (10.2%)	At least 1 month	Below 6 hours: 57 (36.3%) 6–12 hours: 82 (60.5%) > 12 hrs: 18 (11.4%)	None	• Caregiving puts a financial strain on the caregiver • Caregiving is associated with high expenses • ICGs felt unhealthy due to caring work
Gawulayo (2021) [48] South Africa	Qualitative (Not specified)	Family Caregiver assisting with ADL Mean age: 48 years Age range: 33 to 84 years Sex: Female 5 (62.5%) Employment: Employed: 2 (25%); Unemployed: 6 (75%)	8	Stroke	Tertiary: 1 (12.5%) Secondary: 7 (87.5%)	At least 6 months	Not reported	None	• Caregivers were unable to maintain financial stability. • Job challenges like resigning from jobs, becoming self-employed and changing jobs to flexible ones so they are available when needed. • They covered food and transport costs. • They experienced financial difficulties, exhaustion and burnout
Esmaili (2018) [65] Tanzania	Qualitative (Not specified)	Caregiver children with cancer Mean age: Not reported Age range: Not reported Sex: Female 11 (55%) Employment: Not reported	20	Hepatoblastoma (3), Kaposi’s sarcoma (3), Osteosarcoma (3), Wilms tumor (3), Chronic myelogenous leukemia (2), Nephroblastoma (2), Chondroid sarcoma (1), Neuroblastoma (1), Non-Hodgkin lymphoma (1), Retinoblastoma (1)	Not reported	Not reported	Not reported	None	• Caregivers took loans to care for their patient. • They defaulted home rent payments in order to cover patient’s bills. • They requested for early hospital discharge due to low finances, affecting prognosis of their patients
Duru (2021) [40] Nigeria	Quantitative (Health facility outpatients)	Parents of children with Congenital Heart Disease (CHD) Mean age: Not reported Age range: Not reported Sex: Not reported Employment: Not reported	121	Acyanotic congenital heart diseases (79.3%), Cyanotic congenital heart (20.7%)	Mothers 84.3%; Guardians 10.7%; Fathers 5.0%	At least 3 months	Not reported	None	• Mean annual indirect costs by ICG was USD 53.42 ± USD 88.12 and ranged from USD 1.38 to USD 575.00. • Caregivers missed days at work and had reduction in income and productivity. • Mean direct cost of care was USD 240.00 ± 319.52, Range: USD 0.42 to 2123.78. • Total mean cost USD 244.31 ± 333.92, Range: USD 0.42 to USD 2,127.8 • Total spent by the 121 households on care in one year was USD 28,339.80. • 35.5% of the households suffered catastrophic health expenditure (CHE) due to healthcare expenses of the sick child. • Families had to cut down on basic needs like education, feeding, clothing and compromise living standards to cope with the long-term cost of healthcare.
Dhada (2019) [49] South Africa	Qualitative (Health facility outpatients)	ICGs of children Mean age: 38 years Age range: 20 to 67 years Sex: Female 14 (100%) Employment: Employed: 2 (14%); Unemployed: 12 (86%)	14	Diabetes Mellitus	Mother: 9 (64%) Aunt: 3 (22%) Grandmother: 2 (14%)	• The mean duration of each caregiver’s experience of caring for a child with DM was 3.4 (±2.6) years, with a range between 5 months and 10 years • Diabetes caregiving years (total): 47.4	Not reported	None	• They experienced stress, fear and anxieties especially in the initial stages of care. • They could not continue with their jobs/work. • Purchased medical items at high costs. • Anxiety over where they will get the needed money. • Increased financial strain on the family.
Dawson (2020) [59] Ghana	Quantitative (Health facility inpatients)	Primary family caregivers Mean age: 40years Age range: <30 to >50 years Sex: Female 120 (76.9%) Employment: Employed: 130 (83.3%) Unemployed: 26 (16.7%)	156	Lymphoma	Auntie/Uncle: 13 (8.3%) Parent: 108 (69.2%) Grandparent: 19 (12.2%) Sibling: 16 (10.3%)	at least 1 month	Mean care hours/ month 52.3 About 56.8 and 54.0 hours per month are spent by carers in the private sector and self-employed (respectively) assisting patients with daily activities. Average time spent by those unemployed is 53.3 hours.	Cost-Of-Illness Analysis ZBI EUROHIS QoL	• Average caregiving cost per month of caregiving for lymphoma a patient was estimated at US$440.32 (SD: US$205.75) with 97% of the total cost constituting direct cost [US$427.05 (SD: US$203.92)]. • Caregivers raised 58% of the direct costs, and the rest came from contributions from relatives, gifts and donations and borrowing • Caregivers with high caregiver burden had lower quality of life (EUROHIS-QOL)
Bessa (2012) [50] Togo	Qualitative (Not specified)	ICGs Mean age: 43 years Age range: 30 to 65 years Sex: Female: 14 (82%) Employment: Employed: 7 (41%) Unemployed: 10 (59%)	17	Cancer (Not specified)	Siblings: 5 (29%) Spouse: 4 (24%) Children: 3 (18%) Parent: 2 (12%) Relatives: 2 (12%) Friends: 1 (6%)	9 (53%) had been caring for a loved one between 4 and 12 months prior to the study	Not reported	None	• Caregivers spent about $66 monthly (range = $21 –$213) on the loved one with cancer. • They developed sicknesses like back pain • 47% participants found difficulty maintaining balance between paid work and caregiving. • Forced to leave paid work because there isn’t time causing financial challenges. • They found it difficult to concentrate at work and had reduced work efficiency. • Absenteeism at work. • Failure to pay medical bills led to debts and taking incomplete doses for the patients. for Unable to pay fully for their patient’s • They experienced emotional distress and psychological challenges • Loss of will to live and hope. • Caregivers’ children have had to drop out of school because she is not home to support children to go to school.
BeLue (2018) [56] Senegal	Qualitative (Health facility outpatients)	Family caregiver Mean age: 39.7 years Age range: 20 to 92 years Sex: Female: 10 (52.6%) Employment: Not reported	19	Diabetes mellitus	Children: 7 (36.8%) Spouse: 4 (21.1%) Sibling: 2 (10.5%) Others: 6 (31.6%)	Not reported	Not reported	None	• Stress and exhaustion from care work. • Difficult to maintain work and caregiving balance. • Caregivers adopted healthy lifestyles like healthy meals, regular exercises, regular medical checkups. • Improved their knowledge on diabetes and its management
Bekui (2023) [66] Ghana	Qualitative (Not specified)	Family caregiver of a child below 12 years Mean age: 39.7 years Age range: 30 to 69 years Sex: Female 3 (20%) Employment: Employed: 12 (80%) Unemployed: 3 (20%)	15	Childhood cancers (Not specified)	Not reported	at least 3 months	Not reported	None	• Economic • Depletion of the caregivers’ savings due to the cost while in the hospital. • Caregivers got loans and sold off personal property to raise money for treatment. • Some temporarily left work to make time for their sick children. • Loss of employment: Others gave up their businesses or employment in order to take care of their sick children. Health • Physical health challenges eg hypertension, backache, knee pains, headaches, sharp pains in the heart, fatigue • Psychological challenges resulting into loss of appetite, altered eating patterns and weight loss. • They missed their meals. • Sleep disruption in response to actual and anticipated care demand and child’s condition
Akpan-Idiok (2014) [41] Nigeria	Quantitative (Not specified)	ICGs Mean age: 35.9 years Age range: <30 to 70 years Sex: Female 132 (62.9%) Employment: Employed 69 (32.9%) Unemployed: 141 (67.1%)	210	Cancer (Not specified)	Children: 132 (62.9%) Spouse/partner: 43 (20.5%) Sibling: 21 (10%) Friend: 14 (6.7%)	Not reported	Not reported	Zarit Burden Interview (ZBI)	41.4% of the caregivers experienced financial burden
Adejoh (2021) [9] Nigeria, Uganda, & Zimbabwe	Qualitative (Not specified)	ICGs Mean age: 37 years Age range: 19 to 75 years Sex: Female 24 (50%) Employment: Not reported	48	Cancer (Not specified)	Sibling 18 (37.50%) Children: 15 (31.25%) Spouse: 11 (22.92%) Parent 5 (8.33%)	Not reported	Not reported	None	• They gave up jobs to provide care • Absenteeism at work to keep hospital appointment. • They had to reprioritize their resources as they put most resources in treatment of the patient at the expense of other vital things such as house rent.
Abba (2022) [42] Nigeria	Quantitative (Health facility outpatients)	ICGs Mean age: 28.1 years Age range: 18 to 57 years Sex: Female 132 (60.6%) Employment: Unemployed 83 (38.1%) Employed 135 (61.9%)	218	Stroke	Not reported	1–26 weeks: 204 (93.6%) 27–52 weeks: 12 (5.5%) 53–78 weeks: 1 (0.5%) 79–104 weeks: 1 (0.5%)	Full-time: 139 (63.8%) Part-time: 79 (36.2%)	None	• The overall prevalence of musculoskeletal pain among informal caregivers of stroke survivors in the past 12 months of caring was 16.5%. • They had pains in the neck, shoulder, elbow, wrist, upper back, low back, hips, knee, and ankle.
Yousif (2022) [53] Sudan	Quantitative (Not specified)	Parents of children with diabetes Mean age: Not reported Sex: Not reported Employment: Not reported	150	Diabetes Mellitus	Fathers and mothers (Proportion not given)	Not reported	Not reported	Depression, Anxiety, Stress Scale-21 Items (DASS-21)	• Patterns of psychological problems of parents of children with diabetes • Depression level • Mild: 41 (27.3%) • Moderate: 34 (22.7%) • Severe: 4 (2.7%) • Extremely severe: 3 (2.0%) • Anxiety level • Mild: 37 (24.6%) • Moderate: 10 (6.7%) • Severe: 7 (6.5%) • Extremely severe: 10 (11.2%) • Stress level • Mild: 36 (24.0%) • Moderate: 22 (14.7%) • Severe: 12 (8.0%) • Extremely severe: 2 (1.3%)
Ogunyemi (2021) [43] Nigeria	Quantitative (Not specified)	Primary ICGs Mean age: Not reported Age range: ≤18 to 60 years Sex: Female 223 (57.9%) Employment: Employed: 231 (60%) Unemployed: 154 (40%)	385	Cancer (Not specified)	Parent: 133 (34.6%) Sibling: 93 (24.2%) Spouse: 61 (15.8%) Child: 32 (8.3%) Others: 66 (17.1%)	≤ 6 months: 125 (43.4%) 7–12 months: 123 (42.7%) ≥ 13 months: 40 (13.9%) Median = 12 (±12.6)	1–5 hours: 204 (53.0%) 6–11 hours: 132 (34.3%) ≥ 12 hours: 49 (12.7%) Median = 6 (±5.8)	Zarit Burden Interview (ZBI)	Financial burden: • 42.3% caregivers experienced moderate financial burden. • 47.3% caregivers experienced severe financial burden
Malangwa (2022) [62] Tanzania	Quantitative (Outpatients and inpatients)	Caregivers with children confirmed with cancer. Mean age: Not reported Age range: 18–60 years Sex: Female 70 (72.9%) Employment: Employed: 44 (45.8%) Unemployed: 52 (54.2%)	96	Cancer (Not specified)	Parent: 79 (82.3%) Siblings: 8 (8.3%) Aunt/Uncle: 3 (3.1%) Grandparent: 6 (6.3%)	Not reported	Not reported	Hopkins Symptoms Checklist (HSCL-25) tool.	The prevalence of psychological distress among caregivers of children receiving cancer treatment was 66.7% of the study population using HSCL-25.
Ababacar (2022) [67] Senegal	Quantitative (Not specified)	ICGs Mean age: 42.6 Age range: 22 to 75 years Sex: Female 32 (54.2%) Employment: Employed: 42 (70%) Unemployed: 18 (30%)	60	Stroke	Children (50%) Spouses (25%) Siblings (13.33%) Nephews/nieces (11.66%)	Not reported	Not reported	Zarit Burden Interview (ZBI)	• Mean duration of work stoppage of caregiver was 23.86 days (SD 64.53 days) with extremes ranging from 0–365 days. • The average cost of financial losses related to the work stoppage of the main caregiver was 206.92 USD (SD 583.16 USD) with extremes of 0 and 3103.44 USD. • Among the employed caregivers, 47.61% changed jobs, 28.57% changed work schedule and 23.80% stopped working. • 45% caregivers experienced mild to moderate burden of care as assessed using ZARIT score. The mean ZARIT score was 2.65 (standard deviation 1.52) with extremes of 0 and 5.5.
Gosse (2024) [68] Tanzania	Qualitative (Outpatients and inpatients)	Male partners of women with cervical cancer Mean age: Not reported Age range: 41 to 80 years Sex: Male 13 (100%) Employment: Employed 11 (84.6%), Unemployed 2 (15.4%)	13	Cervical cancer	Male partner 13 (100%)	1 year 4 (30.8%) 2 years 5 (38.4%) ≥ 3 years 4 (30.8%)	Not reported	None	The caregivers experienced: • Psychological distress and worries • Financial hardship due to increased financial demands and high costs. • Disruption of economic activities. • Selling of personal/family assets
Najjuka (2023) [69] Uganda	Qualitative (Not specified)	Family member of cancer patient Mean age: 32.5 years Age range: 19–49 years Sex: Female 6 (50%) Employment: Not reported	12	Various cancers (at stage 3 and 4)	Child: 7 (58.3%) Parent: 3 (25%) Spouse: 1 (8.3%) Niece/Nephew: 1 (8.3%)	3–11 months: 2 (16.7%) 1–2 years: 8 (66.7%) > 2 years: 2 (16.7%)		None	• Financial drain • Physical health problems like headache, chest pain, fatigue and body pain • Sleep disturbances • Psychological challenges, including stress, fear, sadness and worry. • Absenteeism at work • Conflicts at work

Note: All data was extracted by Ephraim Kisangala from January to March 2023 and in May 2024.

ICGs—Informal caregivers.

### Quality of the included studies

Overall, the included studies were of good quality as shown in S1 Table. More than three-quarters (85%) of these studies met at least four of the five criteria. In two studies [37,67], only two of the five criteria were met. The weaknesses of the quantitative studies were predominantly related to the failure to report or justify the sampling strategy, sample size and risk of nonresponse bias. All the qualitative studies met at least four of the Mixed Methods Appraisal Tool (MMAT) criteria except one [29] where three of the five criteria were met.

### Participants characteristics

There were 4185 participants in this review, majority of whom were either parents, siblings, children, spouses or other relatives of the patients and with an age range of ten [35] to ninety-two years [56]. The proportion of non-familial caregivers was less than 10% in each of the seven studies [31,36,38,41,47,50,61] where they were part of the sampled populations. In 19.1% (9/47) of the studies [12,40,49,53,58,62,64–66], the participants were caregivers of children with chronic diseases. More than half of the studies (53.1%, 25/47) focused on caregivers of people with cancers [9,11,12,25–28,33,35,37,41,43,47,50,57–66,70]. The participants in the remaining studies were caregivers of people with cardiovascular diseases [21,29–32,34,36,39,40,42,44,46,48,51,54,55,67] (36.2%, 17/47) and diabetes mellitus [38,49,53,56] (8.5%, 4/47). There was no study with caregivers of people with chronic obstructive pulmonary disease. Generally, there were more female participants than male participants. There were only male participants in one study [68] and the proportion of female participants did not reach half in just five (10.6%) of the included studies [33,39,57,63,66]. The characteristics of the included studies and participants are summarised in **Table 1**.

Only two (4.3%) of the 47 studies had paid caregivers, with their number being a very small proportion of the respective study participants, that is 8.8% (7/80) in one study [31] and 1% (1/100) in another [38]. Similarly, three out of twenty participants in another study were not actively involved in care because their patients had died [25,26]. There was no study with a comparison group. Also, authors of ten studies [33,34,36,38,42,51,54,57,60,62] (41.7% of the quantitative studies) explained that they excluded caregivers who had any of the potential negative outcomes of caring being investigated in their respective studies prior to becoming a carer. For example, a study investigating the impact of caregiving on the mental health of caregivers excluded participants who had a diagnosis of a mental health disease prior to starting the provision of informal care.

### Activities performed by the caregivers

The roles performed by caregivers were described in two-fifths (20/47) of the included studies. With the exception of one [47], these studies listed the various things caregivers did. In this particular study, the authors simply stated that performed activities of daily living were demanding and exhausting but did not specify what tasks they were.

In this review, we have grouped informal caregivers’ activities into three activities.

i. Basic activities of daily living

These are personal care tasks that are important for one’s everyday living. The caregivers played a critical role in supporting care recipients with personal care activities like bathing and dressing [9,11,21,28,29,39,44,45,50,55,63,69], toileting [21,50] and feeding [21,29,66,69]. They also assisted with functional mobility activities that support the patient to move in and out of bed, repositioning, ambulating and moving up/down the stairs [21,29,39,44,50,69].

ii. Instrumental activities of daily living

These activities were quite diverse and included caring for the patient’s children [44], shopping for the patient [11,55] and doing domestic tasks (house chores) like room cleaning, making meals, laundry and washing utensils [9,11,28,29,40,44,48,50,56,63,69]. The caregivers were actively involved in the health management of their sick loved ones by managing medical appointments and transporting the patient to hospital [9,11,21,25,27,29,40,48,49,55,56,58,66,69], communicating with health workers on the patient’s behalf [9,69], paying medical expenses [9,21,25,39,40,49,50,56,58,65,69], providing company and psychological support to patient [9,21,56,58] and monitoring the patient’s symptoms [11,49,63].

iii. Medical activities

The caregivers performed activities that would ideally require a certain level of medical training, experience or knowledge. Nine studies [9,11,21,29,44,45,56,63,69] highlighted specific medical procedures that were done by the caregivers. These included changing colostomy bags, wound dressing, helping patient use oxygen breathing apparatus and administering both oral and injectable medications for the patient. Despite performing these tasks, the caregivers acknowledged their lack of knowledge and skills. This was clearly articulated by one caregiver: “*To be honest I cannot say I have been doing a great job of caring for my father*,*…it is just a ‘trial and error’ situation*. *It feels like I am not doing enough*. *Somehow*, *I feel like giving up…*’[47].

### Reasons/Motivation to provide care

The reasons for taking up caregiving roles were recorded in six (12.2%) studies [11,21,29,47,50,66], all of which were qualitative. From their submissions, it was clear that the decision to provide informal care depended on two things; how related or connected the caregiver was to the patient and the caregiver’s level of responsibility (social obligation) over the patient.

i. Relationship between the caregiver and the patient

Regarding the relationship (or connectedness) to the patient, caregivers narrated how they provided care because of the love that existed in their relationship either as family members, neighbours or friends [21,50]. Because of this bond (and feeling of belonging), they willingly volunteered and often considered themselves the most suitable persons to care for their loved ones. The closeness of the relationship between the caregiver and recipient is a strong motivator for becoming a caregiver. This view was echoed by a Togolese caregiver who was quoted saying, “*The problem is that nobody can take care of my mother as I am doing it*. *If something does not belong to you*, *you treat it differently*.” Another participant in the same study added, “*I see myself in a better position to do the care work because I know her very well*.” [50].

ii. Level of responsibility the caregiver has over the patient.

Quite relatedly, the caregivers also viewed their decision to care as a form of responsibility or a social obligation [11,29,47,50,66]. They naturally took on the role and were as committed in the work as they would in any other professional job. They considered caring as a duty they were obliged to perform against all odds for those they were socially expected to support. One mother explained how she had to take her older child (with cancer) to the hospital despite her own poor health: “*I remember there was a time I sat in a chair by the bedside throughout the night and my legs got swollen…it was not easy*, *but I passed through all that*. *When I gave birth on Thursday*, *the following Tuesday I had to bring my sick son to the hospital for review…I had to do it*.” [66]

### Impact of caregiving on the caregivers

All studies had an aspect of the impact (health or economic) caregiving had on the carers. In this section, results on how the impact was measured, positive impact of caregiving, economic impact of caregiving and health impact of caregiving was provided. These are also summarised in **Table 2.**

**Table 2:** Summary of caregiver activities, motivations and the impact of caregiving on the caregivers.

Results	Sub-heading	Studies
Activities done by caregivers (20 studies)	Basic activities of daily living	[9,11,21,28,29,39,44,45,50,55,63,66,69]
	Instrumental activities of daily living	[9,11,21,25,27–29,39,40,44,45,48–50,55,56,58,63,65,66,69]
Motivation to become a caregiver	Relationship between the caregiver and the patient	[21,50]
	Level of responsibility the caregiver has over the patient	[11,29,47,50,66]
Impact of caregiving	Positive impact of caregiving on caregivers	[45,56,69]
	Economic impact of caregiving	1. Direct cost of caregiving [40,50,59,67] 2. Social cost of caregiving [9,29,44,65,69] 3. Work-related effects of caregiving [9,11,12,21,25,29,30,44–50,52,55,56,58,63,66,67,69] 4. Financial challenges [11,25,30,31,35,37,39–41,43,44,47–49,52,55,58,59,63,65,66,68,69]
	Health impact of caregiving	1. Impact on the physical health of the caregivers [21,26,28,29,34,39,42,44,47,50,52,66,69] 2. Impact on the mental health of the caregivers [21,26,29,32,33,36,38,44,46,47,49–51,53,54,56–58,60–62,64,67–69]

#### Measurement of the impact of caregiving

From the findings, about half of the studies (22/47) used standardised tools/instruments to determine the impact of caregiving. It was only the qualitative studies and one quantitative study [40] where no standardised instrument was used in measuring the impact of caregiving. The tools (in their original or modified form) used include, the Zarit Burden Interview (ZBI) questionnaire [33,35,36,38,39,41,43,54,59,67], the Hospital Anxiety and Depression Scale (HADS) [32,54,60,61], Caregiver strain index (CSI) [30,31,39,52], Nordic Musculoskeletal Questionnaire (NMQ) [34,42], General health questionnaire (GHQ) [33,37,38], Patient Health Questionnaire-9 (PHQ-9) for Depression [57] Caregiver Reaction Assessment scale (CRA) [60], Beck’s depression inventory (BDI) scale [64], Shona Symptoms Questionnaire (SSQ) [51], The modified Rankin scale (mRS) [54], Depression, Anxiety, Stress Scale-21 Items (DASS-21) [53] EUROHIS QoL [59], Modified version of the Frankfurter Befindlichkeit Skala (FBS) [37] and Hopkins Symptom Checklist (HSCL-25) tool [62]. Two studies [40,59] used cost of illness approaches to estimate the direct and indirect costs incurred by the caregivers who supported people with chronic diseases.

#### Positive impact of caregiving

Although the focus of most studies was on the negative impact of caregiving, three [45,56,69] in particular had caregivers who described how rewarding the activity was to them. They commented on how the situation strengthened the bond with the person being cared for, thus enabling them to develop the dedication and emotional strength to continue caring. Furthermore, the practice of caring became a strong motivation for improvement of their own health [56]. Whenever they saw how their loved ones suffered, they felt energized to do whatever was necessary to avoid getting the same disease. They became motivated to adopt a healthier lifestyle that included having regular medical check-ups, doing more exercises and taking healthier meals.

Because of their involvement in the day-to-day management of the care recipients, they acquired skills and knowledge to protect themselves from acquiring the disease and to fulfil the care needs better. For example, one caregiver who was always there whenever the doctor came to treat his wife stated that, “*my experience helps a lot to avoid diabetes in my own life*. *What the doctor says to my wife*, *I also apply to my own life because that is good for everyone*, *not just the diabetic patients*.” [56]

#### Economic impact of caregiving

There were three key findings under economic impact, and these were the cost of caregiving, the work-related consequences of caregiving and the subsequent financial challenges arising from caregiving.

- Direct costs of caregiving
  Surprisingly, only four studies [40,50,59,67] provided information on the monetary cost of caregiving on the caregiver. There was no uniformity on what constituted the caregiving cost or how it was reported. For example, the total direct costs were reported in two studies [40,59], total indirect costs in three studies [40,59,67] and financial loss caused by the main caregiver’s withdrawal from employment in two studies [40,67]. Furthermore, two studies presented their findings using average monthly costs [50,59] while the remaining two used average annual costs [40,67]. In one study, the total cost of caregiving [USD 66 (Range: 21–213) per month] was only stated, and no description or explanation on how the amount was estimated was provided [50]. While reporting the different costs, standard deviation was provided in three studies [40,59,67], range in two [40,50] and median in just one study [59]. The cost of caregiving varied significantly both within and across the studies. The study [40] with the highest average caregiving cost at USD 244.31 (SD: USD 333.92, Range: USD 0.42 ‐ USD 2127.8) per year was conducted among caregivers of children with congenital heart disease in Nigeria while the study among caregivers of children with lymphoma in Ghana had the lowest reported cost at USD 440.32 (SD: USD 265.75, Median: USD 409.60) per month [59]. The average annual productivity loss caused by the main caregiver’s employment cessation ranged from USD 53.42 (SD: USD 88.12, Range: USD 1.38 ‐ USD 575.00) [40] to 206.92 USD (SD: USD 583.16, Range: 0 to 3103.44 USD) [67]. The direct cost of caregiving made the biggest proportion of caregiving expenditure, accounting for nearly 97% of the total costs in one of the studies [59].
- Social cost of caregiving
  Of note, are the 5/47 studies [9,29,44,65,69] that commented on the social cost associated with informal caregiving. As one caregiver put it, caregiving “*restricts our lives*” [44] giving them little room for engagement in other activities. A female caregiver in Cameroon commented on the cost involved in letting go of her previously planned activities. She stated, “*Since my mother is in this state*, *[…] I am obliged to suspend my participation in the church every morning as I did before because it is no longer possible*! *I had to review all my activities because I had to find time for my mother!* *It’s painful but I do it!*” [29].
  While some caregivers had to let go of their regular leisure activities [29], others especially the students ended up missing attending classes at school [44,69].
- Work-related consequences of caregiving
  In 22 (46.8%) studies, there is a clear demonstration of how caregiving affected all aspects of one’s work life (regular source of income). Since caring often required one’s physical presence, the caregivers not only used up all the available work leave (including taking leave without pay) but they were also frequently absent (without permission) from work. This resulted into being queried, warned, sanctioned and in other cases dismissed [9,11,50,63,66,69]. In the study by Ababacar et al. [67] the caregivers were off work for an average of 23.86 days (SD 64.53 days) per year with extremes ranging from 0 to 365 days.
  The participants admitted that they struggled to maintain a balance between work and caregiving roles [9,11,21,45,50,55,56,63,69]. This made them less efficient (productive) at work and unable to meet several deadlines because they frequently arrived late for work, spent less time doing work and struggled to concentrate while at work [12,25,29,50,63]. This situation was well explained by the caregiver who said, “*There was a piece job that I had in Durban*, *it was affected because I had to be here in Pietermaritzburg to take care of her [my mother]*. *I couldn’t stay [at work] for the week and come back on the weekend or month end*, *I had to keep coming back here [to check on my mother]*.” [25]
  As such, they had to make work-related adjustments that would allow them to attend to their sick loved ones too. Part of the adjustments included getting a flexible work schedule that allowed them to work either at night or from home [30,46,52,67] and changing to temporary self-employment or to jobs that are flexible and closer to the patient’s home [25,48,67]. In cases where the caregivers could not maintain both work and caregiving roles, they either voluntarily resigned or were dismissed from work [9,11,12,21,25,44,45,47–50,58,63,66,67,69].
- Financial challenges arising from caregiving.
  There were 23 (48.9%) studies in which caregivers reported experiencing financial challenges. First, they stated that they lost money and their sources of income through covering high medical expenses and loss of jobs [25,35,37,40,47,65,66,68,69]. Their response to such challenges included taking loans to get money to care for the patient [37,59,63,65,66], seeking additional financial support from relatives and institutions like churches [58,59], sale of family properties [58,63,66,68] and requesting for premature discharge from the hospitals [65].
  In cases where resources to cover the expenses were inadequate, the caregivers compromised their standard of living, forewent paying house rent, paying their children’s school fees as well as buying food and clothes in order to meet their patient’s care needs [9,65]. As a result, some were reportedly thrown out of their house due to non-paid rent.
  Overall, caregivers felt that caring was financially burdensome causing them to be in a much less satisfactory financial status than before taking up the role [11,25,30,31,39,41,43,44,48,49,52,55,63]. They described their financial situation as being unstable, strenuous and hard, and they often became anxious whenever they thought about where to get more money to continue with their role [39,48,49]. Quantitatively, the prevalence of caregivers experiencing financial strain ranged from 37.9% to 100% [30,31,41,52].

#### Health Impact of caregiving

Caregiving affected the physical health and mental health of the carers as stated below.

i. Physical health consequences of caregiving

This was found in thirteen studies [21,26,28,29,34,39,42,44,47,50,52,66,69]. The development of new medical conditions and or worsening of existing health conditions among the caregivers affected their physical health and reduced their ability to adequately care [39,66]. Some attributed this to lowering of the body’s immunity [44] that occurred while providing care. They stated that they developed new medical conditions such as hypertension, headache, body pains and swelling of the feet during the period they provided care to the patient [21,47,66,69]. One caregiver even narrated a life-threatening incident that necessitated hospitalization “*…after collapsing while fetching water for the sick son*” [66]. There were also reports of the worsening of the caregivers’ own health condition [21,28,34,42,47,50,66]. The studies show how those with musculoskeletal complications such as pain in upper and lower back, pain in different joints (shoulder, wrist, hips, buttocks, knees, ankle and elbow joint), and pain in the thighs, neck and feet complained of deteriorating health. This was well articulated by a caregiver whose waist problem worsened while caring for her husband. She stated, “*Caring for my husband is not easy; I have a waist problem*, *and I find myself always very tired*. *The waist problem was there but was not as serious as it has become now*, *all because of the role I currently play*” [28].

The caregivers in eleven studies experienced significant lifestyle changes during their work. They felt that their sleep was disturbed because of actual/anticipated care demands, and severity of the condition of the person they cared for [66]. They experienced disruption from sleep, feeling of lack of enough sleep, feeling like they are deprived of sleep as well as short episodes of sleep [26,28,29,47,52,66,69]. Another way caregiving affected their lifestyle was through change in eating habits. This involved either changing the meal time in order to incorporate the caregiving demands/activities, eating the patient’s left over foods or missing the meals due to the lack of money/ time [28,66]. A male carer commented on his changed lifestyle by saying, “*It [life] has changed because I hardly sleep now*, *I just think that I can’t sleep I have to constantly check on him*.” [26]

ii. Mental health consequences of caregiving

The act of caregiving had a profound effect on the mental wellbeing of care providers as stated in half (25) of the studies [21,26,29,32,33,36,38,44,46,47,49–51,53,54,56–58,60–62,64,67–69]. Although most of these studies described the different types of mental health challenges, five [33,38,51,62,67] did not specify as they only used the terms “psychological” and “psychiatric” challenges. Using GHQ [33,38], SSQ [51], HSCL-25 [62] and ZBI [33,38,67] tools, the reported prevalence of psychological/psychiatric challenges among caregivers ranged from 35% to 66.7%.

The caregivers experienced mild to severe forms of depression as was assessed using HADS [32,54,60,61], ZBI [36], PHQ-9 [57], BDI [64] and DASS-21 [53] tools. From these eight quantitative studies, the prevalence of depression ranged from 26% to 72.4%. In addition, there were two qualitative studies with caregivers who reported feeling depressed because of the caregiving work [29,44]. Some got to an extent of being treated for depression as explained by this South African caregiver: “*… I also get sick often*. *I have continuous lung infections from stress*. *I’m on antidepressants as well*.”

Fear, stress and anxiety is another mental health related challenge that caregivers in eleven studies [32,47,49,53,54,56,58,60,61,68,69] reportedly experienced. There was no quantitative paper in which fear or stress was investigated. Instead, five quantitative papers employing HADS [32,54,60,61] and DASS-21 [53] tools found that the prevalence of anxiety among caregivers ranged from 21.6% to 45%. Sometimes, the caregivers thought about the likelihood of acquiring the same illness their loved ones have or death of those they care-for. Such thoughts caused fear, stress and anxiety among the caregivers [47,56]. This challenge was most felt in the initial stages of caring for the patient, when there was a lot of work to be done and when the activities were complex [49]. While one caregiver hinted at how hard some caregiving activities can sometimes be by saying, *“the hardship is just injecting the child part*, *it’s not something that’s easy—it’s not easy–because sometimes you find that the child does not want to be injected…when her sugars are high and you try to inject her–she says ‘you’re injecting me a lot’*.*”*[49]

Another expressed the fear that comes with such activities: *“… The family members you stay with do want to assist with injecting the child but they are scared–so sometimes you’re the only one who’s taking care of the child*.*”* [49]

Five quantitative studies [32,53,54,60,61] explored the prevalence of anxiety among caregivers and found it to be in the range of 21.6% to 45%.

Caregiving was also described as an emotionally challenging activity by participants in five studies [29,46,47,50,68]. They used phrases such as being morally sick, emotionally painful, heartbreaking, emotionally touched, burdens me, disturbed and hopelessness to show their reaction to caring for the sick or the diagnosis given. The extent of suffering that caregivers observed their loved ones go through was enough to create feelings of preferring to “*isolate [oneself] to cry”* and develop thoughts of “*not … living anymore*” [46,50]. Caregivers confessed that such reactions also followed the sudden announcement (by the medical team) of the type disease their loved one was suffering from [29,68]. One participant whose husband had been diagnosed with cancer recalled such a moment: “*It was painful*, *wena [‘you’–exclamation]*. *You … you know that person is a breadwinner and everyone in his family is looking up to him*. *He is supporting everyone*. *Then it was painful*, *but I think that I am recovering now*.” [46]

## Discussion

In this review, we found a total of 4185 participants who participated in 47 qualitative and quantitative studies from across the sub-Saharan African region. These studies were published over a period of more than two decades. This review provides a summary of caregiver activities, motivations, and impact of caregiving on the caregivers in sub-Saharan Africa. It reveals the overwhelming burden healthcare systems place on the caregivers as well as the health, economic and social-cultural aspects of caregiving in the region. As an integral part of a generally underfunded healthcare system in sub-Saharan Africa, informal caregivers need to be supported by their respective governments to have better caregiving experience. Our review adds knowledge to the discussion on informal caregiving in sub-Saharan Africa, and highlights areas which should be prioritised for additional support.

### Reasons of becoming a caregiver

These studies show that a relationship that is strengthened by love and the sense/feeling of responsibility over an individual appear to be the most important factors in the decision to provide care. This finding is consistent with those in other reviews with studies from across the world [17,71]. In our review, children as young as ten years of age also participated in providing care to people with chronic diseases. This can be explained by how responsibilities/activities are shared or delegated in many African multi-generational homes [33,72]. Often, children are assigned the task of providing care for unwell or elderly relatives while adults participate in income generating activities [33,72]. Once assigned the tasks, the children take them as their responsibility. The finding from this and other reviews [17,71] may mean that the reasons for becoming caregivers may not be very different across the world even with varying cultures and traditions.

### Activities performed by caregivers

The caregivers, majority of whom were female and were related to the patient, performed a wide range of activities during their caregiving role. Perhaps the most striking finding is that caregivers did not end at performing the common/regular tasks like support with feeding and with personal care needs, but they did complex activities including changing colostomy bags and administering injectable medication which normally require medical skills/training to do well. This rather interesting finding points to shortage of health workers in most settings in sub-Saharan Africa. In most cases, the caregivers end up performing these tasks when health professional cannot be accessed [73,74]. The implication of such findings is the possibility of caregivers making numerous mistakes while conducting procedures they were not trained to perform and unknowingly endangering themselves and/or those under their care. A recent review conducted in Uganda had findings similar to ours [16]. In their study, the caregivers were mainly females, relatives of the patient and performed most activities as described in our work. The only noticeable difference is that there was no mention of the activities, that we have described as complex. This is possibly because their study either considered diseases (such as HIV) that are rarely complicated by events like surgery which may require changing colostomy bags and wound dressing or the diseases had not yet gotten to a level that required interventions like use of oxygen therapy or administration of injectable medications.

### Impact of caregiving on the caregivers

To measure the impact of caregiving, almost all quantitative studies used a standardised instrument/tool. As shown in the results, a total of 14 different tools were used in the 22 studies to assess the burden of depression, anxiety, stress and financial strain. Even in the studies where the same tool was used, the results were presented differently. For example, in the studies that used Zarit burden Interview (ZBI), two [33,36] reported the overall mean ZBI scores while the rest provided mean scores of each category of burden. This lack of uniformity in the use of measures and presentation of results make it difficult to compare different studies’ results. In this review, ZBI tool was the most used instrument for measuring the impact of caregiving. It was used in 10 out of the 22 studies assessing the health or economic impact using an instrument. This accords with earlier observations from another review which found that ZBI was the most frequently used tool in measuring the burden of caregivers [75].

As discussed in the next three paragraphs, caregiving had both positive and negative effects on the caregivers. As expected, there were more studies focussed on the negative than positive impact of caregiving.

The rewards experienced by informal caregivers in this review can be explained by the post-traumatic growth (PTG) theory which suggests that people facing traumatic or challenging events may experience some positive outcomes from their experience [76]. Caring for people, some of whom are at an advanced stage of a chronic disease can be stressful and traumatic due to the highly demanding nature of the caregiving tasks. Such situations often prompt caregivers to do self-reflection leading to the discovery of new and positive insights as well as renewed purpose for living under the current conditions. Recent studies have used this theory to demonstrate how caregivers’ challenging experiences resulted in positive outcomes like renewed personal strength to continue in their caring role, strengthening of the relationship with care recipients/family members and taking better care of their own health because they better appreciate life [77–79].

As with other reviews [15,16] conducted in sub-Saharan Africa, caregivers with a regular source of income faced work-related challenges such as absenteeism, dismissal from work and low productivity as already listed in the results section. Caregivers’ involvement in extensive physical work like assisting patient with mobility-related activities may result into injuries and exhaustion. Depending on the patient’s care needs and severity of the disease, the caregivers may spend a lot of time with the patient. Under such circumstances, the caregivers will either fail to go to work or arrive late at work leading to reduced productivity and conflicts at work. The exhaustive nature and emotional burden that comes with caring for loved ones also reduces one’s level of concentration and performance while at work. In the end, caregivers are often dismissed or forced to resign from their work. At times, they sought employment in places with work that suited their caregiving responsibilities such as self-employment, temporary employment or jobs with flexible working hours [80]. It is also worth noting that a disrupted career/job has the potential of causing significant financial strain on the career, hinder career progression and affect retirement planning. These are likely to persist even after they are no longer providing care.

As would be expected, informal caregivers experienced both physical health and mental health challenges during their work. Several reviews have had similar findings published [14–17]. This is consistent with allostatic load theory, in which McEwen and Stellar observed that although stressors usually resulted in a normal physiological response as a body’s protective mechanism, exposure to high and sustained stress such as that experienced by informal caregivers caused an abnormal response that resulted in cumulative wear and tear of the body [81]. The allostatic load experienced by caregivers arises from exhaustive physical activities they do, emotional challenges caused by the patient’s condition and logistical stressors like financial strain. Part of the abnormal response includes change in the production and levels of hormones like cortisol and adrenaline which could lead to cardiovascular diseases, weakened immune system, inflammation, metabolic changes (affecting an individual’s appetite) and mental health diseases.

### Limitations of the review

This review had some limitations. First, the second reviewer could only screen a proportion of studies during the title and abstract screening stage and during the full text screening. Also, a single author extracted the data from the included studies with the second reviewer checking the accuracy of the data. A study showed that a complete dual review significantly minimised random errors compared to either single review or limited dual review [82].

Secondly, we were unable to get all the full-texts of the conference abstracts that were included in this review. While the contacts for some authors could not be retrieved, the majority of those contacted either did not respond or did not have the full-texts for their abstracts available. It is therefore possible that some important studies could have been missed.

Furthermore, some studies did not provide adequate information as some data was either unclear or not reported. This includes information on sex, age and well as the impact of caregiving. This limited our ability to extract all the relevant data required to comprehensively analyse the data.

Lastly, most quantitative studies used a cross-sectional study design making it difficult to assess the cause-and-effect relationship, and to understand the changes that occur over time. However, almost half of these studies excluded participants with outcomes of interest prior to starting caregiving.

### Strengths of the review

Part of the strength of this review is in the fact that we used a very broad search strategy to extensively search a good number of relevant databases. We also included non-English studies. These enabled us to capture as many relevant studies and information as possible. The use of a second reviewer improved the reliability and quality of the findings through reducing the errors and biases that could have risen if only one reviewer was involved in the review process.

## Conclusions

Informal caregivers occupy an essential place in the health care system of most sub-Saharan African countries and they perform various tasks, some of which they are ill-prepared or untrained to do. The growing demand for caregivers created by the increasing chronic diseases in the African continent means that health care systems would function better if they are adjusted to prepare caregivers for the caregiving roles, and to support the physical and mental wellbeing of caregivers. Future research should investigate the most suitable tools/instruments for assessing the impact of care on the caregiver.

## Supporting information

S1 Checklist PRISMA checklist. (DOCX)

S1 File Search strategy used in the selected databases. (DOCX)

S1 Table Table showing the quality appraisal of the reviewed articles. (DOCX)

S2 Table Table showing all studies identified in the literature search. (XLSX)
