# Supplementary material for: Health and economic impact of caregiving on informal caregivers of people with chronic diseases in sub-Saharan Africa: A systematic review
Source: PLOS Glob Public Health. 2024 Dec 31;4(12):e0004061. doi: 10.1371/journal.pgph.0004061 (PMC11687699; doi:10.1371/journal.pgph.0004061)
Supplement: S1 File — (DOCX) [file pgph.0004061.s002.docx]

## S1 File. Databases search

Health and economic impact of caregiving on informal caregivers of people with chronic diseases in Sub-Saharan Africa: A systematic review

**Databases**
**Ovid MEDLINE(R) ALL**

| **#** | **Query** | **Results from 1946 to 24 May 2024** |
| --- | --- | --- |
| 1 | exp Caregivers/ | 53,166 |
| 2 | (Informal caregiv* or carer or caregiv* or family caregiv*).mp. [mp=title, book title, abstract, original title, name of substance word, subject heading word, floating sub-heading word, keyword heading word, organism supplementary concept word, protocol supplementary concept word, rare disease supplementary concept word, unique identifier, synonyms, population supplementary concept word, anatomy supplementary concept word] | 123,951 |
| 3 | 1 or 2 | 123,951 |
| 4 | exp Chronic Disease/ | 644,089 |
| 5 | (chronic disease or noncommunicable disease or NCD or Cardiovascular disease or coronary heart disease or Hypertension or cardiovascular patients or myocardial infarction or coronary artery disease or stroke or Ischemic heart disease or Cerebrovascular or Vascular disease or diabetes mellitus or Non-insulin Dependent Diabetes Mellitus or Diabetes or metabolic syndrome or Cancer or tumour or carcino* or leukaemia or lymphoma or melanoma or sarcoma or dysplasia or Hodgkin* or maligna* or metasta* or neoplasm or myeloma or blastoma or COPD or Chronic Obstructive Pulmonary Disease or chronic bronchitis or emphysema or chronic airflow obstruction or pulmonary disease).mp. [mp=title, book title, abstract, original title, name of substance word, subject heading word, floating sub-heading word, keyword heading word, organism supplementary concept word, protocol supplementary concept word, rare disease supplementary concept word, unique identifier, synonyms, population supplementary concept word, anatomy supplementary concept word] | 6,558,131 |
| 6 | exp Noncommunicable Diseases/ | 3,561 |
| 7 | 4 or 5 or 6 | 6,767,849 |
| 8 | exp "Africa South of the Sahara"/ | 269,469 |
| 9 | (sub-Saharan Africa or Africa* or Africa, south of the Sahara or Angola or Benin or Botswana or Burkina Faso or Burundi or Cameroon or Cape Verde or Central African Republic or Chad or Comoros or Congo or Cote d'Ivoire or Djibouti or Eritrea or Ethiopia or Gabon or Gambia or Ghana or Guinea or Kenya or Lesotho or Liberia or Madagascar or Malawi or Mali or Mauritania or Mauritius or Mozambique or Namibia or Niger or Nigeria or Rwanda or Sao Tome or Principe or Senegal or Seychelles or Sierra Leone or Somalia or South Africa or South Sudan or Sudan or Swaziland or Tanzania or Togo or Uganda or Zambia or Zimbabwe).mp. [mp=title, book title, abstract, original title, name of substance word, subject heading word, floating sub-heading word, keyword heading word, organism supplementary concept word, protocol supplementary concept word, rare disease supplementary concept word, unique identifier, synonyms] | 756,645 |
| 10 | 8 or 9 | 756,861 |
| 11 | 3 and 7 and 10 | 789 |

**Database: Embase Classic+Embase**

| **#** | **Query** | **Results from 1947 to 24 May 2024** |
| --- | --- | --- |
| 1 | exp Caregivers/ | 123,075 |
| 2 | (Informal caregiv* or carer or caregiv* or family caregiv* or care giver*or care giving).mp. [mp=title, abstract, heading word, drug trade name, original title, device manufacturer, drug manufacturer, device trade name, keyword heading word, floating subheading word, candidate term word] | 184,257 |
| 3 | 1 or 2 | 184,257 |
| 4 | exp Chronic Disease/ | 276,487 |
| 5 | (Chronic disease or Noncommunicable disease or NCD or cardiovascular disease or Coronary heart disease or hypertension or cardiovascular patients or myocardial infarction or coronary artery disease or stroke or ischemic heart disease or cerebrovascular vascular disease or diabetes mellitus or Non-insulin Dependent Diabetes Mellitus or Diabetes or metabolic syndrome or cancer or tumour or carcino* or leukaemia or lymphoma or melanoma or sarcoma or dysplasia or Hodgkin* or maligna* or metasta* or neoplasm or myeloma or blastoma or COPD or Chronic Obstructive Pulmonary Disease or chronic bronchitis or emphysema or chronic airflow obstruction or pulmonary disease).mp. [mp=title, abstract, heading word, drug trade name, original title, device manufacturer, drug manufacturer, device trade name, keyword heading word, floating subheading word, candidate term word] | 10,189,047 |
| 6 | exp Noncommunicable Diseases/ | 12,864 |
| 7 | 4 or 5 or 6 | 10,226,269 |
| 8 | exp "Africa South of the Sahara"/ | 343,122 |
| 9 | (sub-Saharan Africa or Africa* or Africa, south of the Sahara or Angola or Benin or Botswana or Burkina Faso or Burundi or Cameroon or Cape Verde or Central African Republic or Chad or Comoros or Congo or Cote d'Ivoire or Djibouti or Eritrea or Ethiopia or Gabon or Gambia or Ghana or Guinea or Kenya or Lesotho or Liberia or Madagascar or Malawi or Mali or Mauritania or Mauritius or Mozambique or Namibia or Niger or Nigeria or Rwanda or Sao Tome or Principe or Senegal or Seychelles or Sierra Leone or Somalia or South Africa or South Sudan or Sudan or Swaziland or Tanzania or Togo or Uganda or Zambia or Zimbabwe).mp. [mp=title, abstract, heading word, drug trade name, original title, device manufacturer, drug manufacturer, device trade name, keyword heading word, floating subheading word, candidate term word] | 948,311 |
| 10 | 8 or 9 | 948,596 |
| 11 | 3 and 7 and 10 | 1,501 |

**Database: APA PsycInfo**

| **#** | **Query** | **From 1806 to May 2024** |
| --- | --- | --- |
| 1 | exp Caregivers/ | **39,402** |
| 2 | (Informal caregiv* or carer or caregiv* or family caregiv* or care giver*or care giving).mp. [mp=title, abstract, heading word, table of contents, key concepts, original title, tests & measures, mesh word] | **87,228** |
| 3 | 1 or 2 | **87,228** |
| 4 | exp Chronic Illness/ | **36,167** |
| 5 | (Chronic disease or Noncommunicable disease or NCD or cardiovascular disease or Coronary heart disease or hypertension or cardiovascular patients or myocardial infarction or coronary artery disease or stroke or ischemic heart disease or cerebrovascular vascular disease or diabetes mellitus or Non-insulin Dependent Diabetes Mellitus or Diabetes or metabolic syndrome or cancer or tumour or carcino* or leukaemia or lymphoma or melanoma or sarcoma or dysplasia or Hodgkin* or maligna* or metasta* or neoplasm or myeloma or blastoma or COPD or Chronic Obstructive Pulmonary Disease or chronic bronchitis or emphysema or chronic airflow obstruction or pulmonary disease).mp. [mp=title, abstract, heading word, table of contents, key concepts, original title, tests & measures, mesh word] | **222,060** |
| 6 | 4 or 5 | **246,252** |
| 7 | (sub-Saharan Africa or Africa* or Africa, south of the Sahara or Angola or Benin or Botswana or Burkina Faso or Burundi or Cameroon or Cape Verde or Central African Republic or Chad or Comoros or Congo or Cote d'Ivoire or Djibouti or Eritrea or Ethiopia or Gabon or Gambia or Ghana or Guinea or Kenya or Lesotho or Liberia or Madagascar or Malawi or Mali or Mauritania or Mauritius or Mozambique or Namibia or Niger or Nigeria or Rwanda or Sao Tome or Principe or Senegal or Seychelles or Sierra Leone or Somalia or South Africa or South Sudan or Sudan or Swaziland or Tanzania or Togo or Uganda or Zambia or Zimbabwe).mp. [mp=title, abstract, heading word, table of contents, key concepts, original title, tests & measures, mesh word] | **140,175** |
| 8 | 3 and 6 and 7 | **321** |

**Web of Science search results**

| **#** | **Query** | **Results from inception to 29 Sep 2022** |
| --- | --- | --- |
| 1 | ((TS=(Informal caregiv* or carer or caregiv* or family caregiv* or care giver*or care giving)) OR TI=(Informal caregiv* or carer or caregiv* or family caregiv* or care giver*or care giving )) OR AB=(Informal caregiv* or carer or caregiv* or family caregiv* or care giver*or care giving) | 151,763 |
| 2 | ((TS=(Chronic disease or Noncommunicable disease or NCD or cardiovascular disease or Coronary heart disease or hypertension or cardiovascular patients or myocardial infarction or coronary artery disease or stroke or ischemic heart disease or cerebrovascular vascular disease or diabetes mellitus or Non-insulin Dependent Diabetes Mellitus or Diabetes or metabolic syndrome or cancer or tumour or carcino* or leukaemia or lymphoma or melanoma or sarcoma or dysplasia or Hodgkin* or maligna* or metasta* or neoplasm or myeloma or blastoma or COPD or Chronic Obstructive Pulmonary Disease or chronic bronchitis or emphysema or chronic airflow obstruction or pulmonary disease)) OR TI=(Chronic disease or Noncommunicable disease or NCD or cardiovascular disease or Coronary heart disease or hypertension or cardiovascular patients or myocardial infarction or coronary artery disease or stroke or ischemic heart disease or cerebrovascular vascular disease or diabetes mellitus or Non-insulin Dependent Diabetes Mellitus or Diabetes or metabolic syndrome or cancer or tumour or carcino* or leukaemia or lymphoma or melanoma or sarcoma or dysplasia or Hodgkin* or maligna* or metasta* or neoplasm or myeloma or blastoma or COPD or Chronic Obstructive Pulmonary Disease or chronic bronchitis or emphysema or chronic airflow obstruction or pulmonary disease)) OR AB=(Chronic disease or Noncommunicable disease or NCD or cardiovascular disease or Coronary heart disease or hypertension or cardiovascular patients or myocardial infarction or coronary artery disease or stroke or ischemic heart disease or cerebrovascular vascular disease or diabetes mellitus or Non-insulin Dependent Diabetes Mellitus or Diabetes or metabolic syndrome or cancer or tumour or carcino* or leukaemia or lymphoma or melanoma or sarcoma or dysplasia or Hodgkin* or maligna* or metasta* or neoplasm or myeloma or blastoma or COPD or Chronic Obstructive Pulmonary Disease or chronic bronchitis or emphysema or chronic airflow obstruction or pulmonary disease) | 8,813,031 |
| 3 | ((TS=(sub-Saharan Africa or Africa* or Africa, south of the Sahara or Angola or Benin or Botswana or Burkina Faso or Burundi or Cameroon or Cape Verde or Central African Republic or Chad or Comoros or Congo or Cote d'Ivoire or Djibouti or Eritrea or Ethiopia or Gabon or Gambia or Ghana or Guinea or Kenya or Lesotho or Liberia or Madagascar or Malawi or Mali or Mauritania or Mauritius or Mozambique or Namibia or Niger or Nigeria or Rwanda or Sao Tome or Principe or Senegal or Seychelles or Sierra Leone or Somalia or South Africa or South Sudan or Sudan or Swaziland or Tanzania or Togo or Uganda or Zambia or Zimbabwe)) OR TI=(sub-Saharan Africa or Africa* or Africa, south of the Sahara or Angola or Benin or Botswana or Burkina Faso or Burundi or Cameroon or Cape Verde or Central African Republic or Chad or Comoros or Congo or Cote d'Ivoire or Djibouti or Eritrea or Ethiopia or Gabon or Gambia or Ghana or Guinea or Kenya or Lesotho or Liberia or Madagascar or Malawi or Mali or Mauritania or Mauritius or Mozambique or Namibia or Niger or Nigeria or Rwanda or Sao Tome or Principe or Senegal or Seychelles or Sierra Leone or Somalia or South Africa or South Sudan or Sudan or Swaziland or Tanzania or Togo or Uganda or Zambia or Zimbabwe)) OR AB=(sub-Saharan Africa or Africa* or Africa, south of the Sahara or Angola or Benin or Botswana or Burkina Faso or Burundi or Cameroon or Cape Verde or Central African Republic or Chad or Comoros or Congo or Cote d'Ivoire or Djibouti or Eritrea or Ethiopia or Gabon or Gambia or Ghana or Guinea or Kenya or Lesotho or Liberia or Madagascar or Malawi or Mali or Mauritania or Mauritius or Mozambique or Namibia or Niger or Nigeria or Rwanda or Sao Tome or Principe or Senegal or Seychelles or Sierra Leone or Somalia or South Africa or South Sudan or Sudan or Swaziland or Tanzania or Togo or Uganda or Zambia or Zimbabwe) | 1,302,618 |
| 4 | #1 AND #2 AND #3 | 1,042 |

**Global Health Database:**

| **#** | **Query** | **Results from 1973 to Week 21, 2024** |
| --- | --- | --- |
| 1 | careproviders.sh. | 9,066 |
| 2 | (Informal caregiv* or carer or caregiv* or family caregiv* or care giver*or care giving).mp. [mp=abstract, title, original title, heading words, cabicodes words] | 16,100 |
| 3 | 1 or 2 | 19,923 |
| 4 | exp chronic diseases/ | 26,840 |
| 5 | exp noncommunicable diseases/ | 2,827 |
| 6 | (Chronic disease or Noncommunicable disease or NCD or cardiovascular disease or Coronary heart disease or hypertension or cardiovascular patients or myocardial infarction or coronary artery disease or stroke or ischemic heart disease or cerebrovascular vascular disease or diabetes mellitus or Non-insulin Dependent Diabetes Mellitus or Diabetes or metabolic syndrome or cancer or tumour or carcino* or leukaemia or lymphoma or melanoma or sarcoma or dysplasia or Hodgkin* or maligna* or metasta* or neoplasm or myeloma or blastoma or COPD or Chronic Obstructive Pulmonary Disease or chronic bronchitis or emphysema or chronic airflow obstruction or pulmonary disease).mp. [mp=abstract, title, original title, heading words, cabicodes words] | 688,628 |
| 7 | 4 or 5 or 6 | 702,121 |
| 8 | exp "Africa South of Sahara"/ | 232,921 |
| 9 | (sub-Saharan Africa or Africa* or Africa, south of the Sahara or Angola or Benin or Botswana or Burkina Faso or Burundi or Cameroon or Cape Verde or Central African Republic or Chad or Comoros or Congo or Cote d'Ivoire or Ivory Coast or Djibouti or Eritrea or Ethiopia or Gabon or Gambia or Ghana or Guinea or Kenya or Lesotho or Liberia or Madagascar or Malawi or Mali or Mauritania or Mauritius or Mozambique or Namibia or Niger or Nigeria or Rwanda or Sao Tome or Principe or Senegal or Seychelles or Sierra Leone or Somalia or South Africa or South Sudan or Sudan or Swaziland or Tanzania or Togo or Uganda or Zambia or Zimbabwe).mp. [mp=abstract, title, original title, heading words, cabicodes words] | 365,965 |
| 10 | 8 or 9 | 365,965 |
| 11 | 3 and 7 and 10 | 312 |

**CINAHL Database**

| **#** | **Query** | **Limiters/Expanders** | **Last Run Via** | **Results from inception to 11 Oct 2022** |
| --- | --- | --- | --- | --- |
| S11 | S3 AND S7 AND S10 | Expanders - Apply equivalent subjects Search modes - Boolean/Phrase | Interface - EBSCOhost Research Databases Search Screen - Advanced Search Database - CINAHL | 986 |
| S10 | S8 OR S9 | Expanders - Apply equivalent subjects Search modes - Boolean/Phrase | Interface - EBSCOhost Research Databases Search Screen - Advanced Search Database - CINAHL | 216,500 |
| S9 | TX sub-Saharan Africa or Africa* or Africa, south of the Sahara or Angola or Benin or Botswana or Burkina Faso or Burundi or Cameroon or Cape Verde or Central African Republic or Chad or Comoros or Congo or Cote d'Ivoire or Djibouti or Eritrea or Ethiopia or Gabon or Gambia or Ghana or Guinea or Kenya or Lesotho or Liberia or Madagascar or Malawi or Mali or Mauritania or Mauritius or Mozambique or Namibia or Niger or Nigeria or Rwanda or Sao Tome or Principe or Senegal or Seychelles or Sierra Leone or Somalia or South Africa or South Sudan or Sudan or Swaziland or Tanzania or Togo or Uganda or Zambia or Zimbabwe | Expanders - Apply equivalent subjects Search modes - Boolean/Phrase | Interface - EBSCOhost Research Databases Search Screen - Advanced Search Database - CINAHL | 216,500 |
| S8 | TX sub-saharan africa | Expanders - Apply equivalent subjects Search modes - Boolean/Phrase | Interface - EBSCOhost Research Databases Search Screen - Advanced Search Database - CINAHL | 9,308 |
| S7 | S4 OR S5 OR S6 | Expanders - Apply equivalent subjects Search modes - Boolean/Phrase | Interface - EBSCOhost Research Databases Search Screen - Advanced Search Database - CINAHL | 1,861,794 |
| S6 | TX Chronic disease or Noncommunicable disease or NCD or cardiovascular disease or Coronary heart disease or hypertension or cardiovascular patients or myocardial infarction or coronary artery disease or stroke or ischemic heart disease or cerebrovascular vascular disease or diabetes mellitus or Non-insulin Dependent Diabetes Mellitus or Diabetes or metabolic syndrome or cancer or tumour or carcino* or leukaemia or lymphoma or melanoma or sarcoma or dysplasia or Hodgkin* or maligna* or metasta* or neoplasm or myeloma or blastoma or COPD or Chronic Obstructive Pulmonary Disease or chronic bronchitis or emphysema or chronic airflow obstruction or pulmonary disease | Expanders - Apply equivalent subjects Search modes - Boolean/Phrase | Interface - EBSCOhost Research Databases Search Screen - Advanced Search Database - CINAHL | 1,861,794 |
| S5 | TX noncommunicable diseases | Expanders - Apply equivalent subjects Search modes - Boolean/Phrase | Interface - EBSCOhost Research Databases Search Screen - Advanced Search Database - CINAHL | 4,823 |
| S4 | TX chronic disease | Expanders - Apply equivalent subjects Search modes - Boolean/Phrase | Interface - EBSCOhost Research Databases Search Screen - Advanced Search Database - CINAHL | 184,314 |
| S3 | S1 OR S2 | Expanders - Apply equivalent subjects Search modes - Boolean/Phrase | Interface - EBSCOhost Research Databases Search Screen - Advanced Search Database - CINAHL | 110,313 |
| S2 | TX Informal caregiv* or carer or caregiv* or family caregiv* or care giver*or care giving | Expanders - Apply equivalent subjects Search modes - Boolean/Phrase | Interface - EBSCOhost Research Databases Search Screen - Advanced Search Database - CINAHL | 110,313 |
| S1 | TX Caregivers | Expanders - Apply equivalent subjects Search modes - Boolean/Phrase | Interface - EBSCOhost Research Databases Search Screen - Advanced Search Database - CINAHL | 96,631 |
