# Supplementary material for: Health and economic impact of caregiving on informal caregivers of people with chronic diseases in sub-Saharan Africa: A systematic review
Source: PLOS Glob Public Health. 2024 Dec 31;4(12):e0004061. doi: 10.1371/journal.pgph.0004061 (PMC11687699; doi:10.1371/journal.pgph.0004061)
Supplement: S1 Table — (DOCX) [file pgph.0004061.s003.docx]

## **S1_Table**

## **S1 Table showing the quality appraisal of the reviewed studies**

| **Author, year** |  |  | **Quality scores** | | | | |
| --- | --- | --- | --- | --- | --- | --- | --- |
|  | **S1** | **S2** |  |  |  |  |  |
| **QUALITATIVE STUDIES** |  |  | **1.1** | **1.2** | **1.3** | **1.4** | **1.5** |
| Walubita (2018) (12) | Yes | Yes | 1 | 1 | 1 | 1 | 1 |
| Thomas (2008) (44) | Yes | Yes | 1 | 1 | 1 | 0 | 1 |
| Tchokote (2020) (29) | Yes | Yes | 1 | 0 | 1 | 1 | 0 |
| Owoo (2022) (28) | Yes | Yes | 1 | 1 | 1 | 1 | 1 |
| Owoo (2022) (27) | Yes | Yes | 1 | 1 | 0 | 1 | 1 |
| Mthembu (2016) (45) | Yes | Yes | 1 | 1 | 1 | 0 | 1 |
| Mlaba (2021) (26) | Yes | Yes | 1 | 1 | 1 | 0 | 1 |
| Mlaba (2020) (25) | Yes | Yes | 1 | 1 | 1 | 1 | 0 |
| Mensah (2021) (63) | Yes | Yes | 1 | 1 | 1 | 1 | 1 |
| Masuku, (2018) (46) | Yes | Yes | 1 | 1 | 1 | 1 | 1 |
| Masika (2020) (58) | Yes | Yes | 1 | 1 | 1 | 1 | 1 |
| Maree (2018) (11) | Yes | Yes | 1 | 1 | 1 | 1 | 1 |
| Khondowe (2007) (55) | Yes | Yes | 1 | 1 | 1 | 1 | 1 |
| Ketlogetswe (2022) (47) | Yes | Yes | 1 | 1 | 1 | 1 | 0 |
| Gertrude (2019) (21) | Yes | Yes | 1 | 1 | 1 | 1 | 1 |
| Gawulayo (2021) (48) | Yes | Yes | 1 | 1 | 1 | 1 | 1 |
| Esmaili (2018) (65) | Yes | Yes | 1 | 1 | 1 | 1 | 0 |
| Dhada (2019) (49) | Yes | Yes | 1 | 1 | 1 | 1 | 1 |
| Bessa (2012) (50) | Yes | Yes | 1 | 1 | 1 | 1 | 1 |
| BeLue (2018) (56) | Yes | Yes | 1 | 1 | 1 | 0 | 1 |
| Bekui (2023) (66) | Yes | Yes | 1 | 1 | 1 | 1 | 1 |
| Adejoh (2021) (9) | Yes | Yes | 1 | 1 | 1 | 1 | 1 |
| Gosse (2024) (68) | Yes | Yes | 1 | 1 | 1 | 1 | 1 |
| Najjuka (2023) (69) | Yes | Yes | 1 | 1 | 1 | 0 | 1 |
|  | | | | | | | |
| **NRT QUANTITATIVE STUDIES** | **S1** | **S2** | **3.1** | **3.2** | **3.3** | **3.4** | **3.5** |
| Serfontein (2019) (30) | Yes | Yes | 1 | 1 | 0 | 1 | 1 |
| Scheffler (2019) (31) | Yes | Yes | 1 | 0 | 1 | 1 | 1 |
|  | | | | | | | |
| **QUANTITATIVE DESCRIPTIVE STUDIES** | **S1** | **S2** | **4.1** | **4.2** | **4.3** | **4.4** | **4.5** |
| Yusuf (2011) (33) | Yes | Yes | 0 | 0 | 1 | 1 | 1 |
| Wassie (2021) (57) | Yes | Yes | 1 | 1 | 1 | 1 | 1 |
| Vincent-Onabajo (2018) (34) | Yes | Yes | 1 | 1 | 1 | 1 | 1 |
| Onyeneho (2021) (35) | Yes | Yes | 1 | 1 | 1 | 1 | 1 |
| Okeke (2020) (36) | Yes | Yes | 1 | 1 | 1 | 1 | 1 |
| Ohaeri (1999) (37) | Yes | Yes | 0 | 0 | 1 | 0 | 1 |
| Ogunmodede (2019) (38) | Yes | Yes | 1 | 1 | 1 | 1 | 1 |
| Muriuki (2023) (52) | Yes | Yes | 1 | 1 | 1 | 1 | 1 |
| Muliira (2019) (60) | Yes | Yes | 0 | 1 | 1 | 0 | 1 |
| Marima (2019) (51) | Yes | Yes | 1 | 1 | 1 | 1 | 1 |
| Kitoko (2022) (54) | Yes | Yes | 1 | 0 | 1 | 0 | 1 |
| Katende (2017) (61) | Yes | Yes | 1 | 0 | 1 | 1 | 1 |
| Jones (2012) (32) | Yes | Yes | 1 | 0 | 1 | 1 | 1 |
| Gbiri (2015) (39) | Yes | Yes | 0 | 0 | 1 | 1 | 1 |
| Duru (2021) (40) | Yes | Yes | 1 | 1 | 1 | 1 | 1 |
| Dawson (2020) (59) | Yes | Yes | 1 | 1 | 1 | 1 | 1 |
| Akpan-Idiok (2014) (41) | Yes | Yes | 1 | 0 | 1 | 0 | 1 |
| Abba (2022) (42) | Yes | Yes | 0 | 1 | 1 | 1 | 1 |
| Yousif (2022) (53) | Yes | Yes | 1 | 0 | 1 | 1 | 1 |
| Ogunyemi (2021) (43) | Yes | Yes | 1 | 1 | 1 | 1 | 1 |
| Malangwa (2022) (62) | Yes | Yes | 1 | 1 | 1 | 1 | 1 |
| Ababacar (2022) (67) | Yes | Yes | 0 | 0 | 1 | 0 | 1 |
|  | | | | | | | |
| **MIXED METHODS STUDIES** | **S1** | **S2** | **5.1** | **5.2** | **5.3** | **5.4** | **5.5** |
| Mekonnen (2020) (64) | Yes | Yes | 1 | 1 | 1 | 0 | 1 |

*MMAT* Mixed Methods Appraisal Tool [*NRT* Non-randomised Trial, *S1*—Are there clear research questions? *S2*—Do the collected data allow to address the research questions? 1.1—Is the qualitative approach appropriate to answer the research question? 1.2—Are the qualitative data collection methods adequate to address the research question? 1.3—Are the findings adequately derived from the data? 1.4—Is the interpretation of results sufficiently substantiated by data? 1.5—Is there coherence between qualitative data sources, collection, analysis and interpretation? 3.1—Are the participants representative of the target population? 3.2—Are measurements appropriate regarding both the outcome and exposure/intervention? 3.3—Are there complete outcome data? 3.4—Are the confounders accounted for in the design and analysis? 3.5—During the study period, is the intervention/exposure administered as intended? 4.1—Is the sampling strategy relevant to address the research question? 4.2—Is the sample representative of the target population? 4.3—Are the measurements appropriate? 4.4—Is the risk of 0nresponse bias low? 4.5—Is the statistical analysis appropriate to answer the research question? 5.1—Is there an adequate rationale for using a mixed methods design to address the research question? 5.2—Are the different components of the study effectively integrated to answer the research question? 5.3—Are the results adequately brought together into overall interpretations? 5.4—Are divergences and inconsistencies between quantitative and qualitative results adequately addressed? 5.5—Do the different components of the study adhere to the quality criteria of each tradition of the methods involved?], *Y* Criteria satisfied, *N* Criteria not satisfied. 1—Criteria satisfied, *0*—Criteria not satisfied
